# Differential Regulation of TCR‐Induced ZFP36 and ZFP36L1 Expression by Cyclosporin A in CD8^+^ T Cells

**DOI:** 10.1002/eji.70085

**Published:** 2025-11-10

**Authors:** Marian Jones Evans, Twm J. Mitchell, Magdalena Zaucha, Georg Petkau, Martin Turner

**Affiliations:** ^1^ Immunology Programme The Babraham Institute, Babraham Research Campus Cambridge UK

**Keywords:** CD8 T cells, T cell activation, T cell signalling, ZFP36, ZFP36L1

## Abstract

CD8^+^ T cells target infected or malignant cells via the production of pro‐inflammatory cytokines and direct target cell killing. Members of the ZFP36‐family of RNA‐binding proteins, ZFP36 and ZFP36L1, regulate these functions in T cells via the regulation of mRNA stability and protein translation. We investigate the regulation of ZFP36 and ZFP36L1 expression using *in vitro* differentiated OT1 TCR transgenic memory‐like T cells. We characterise the differential kinetics and sensitivity of ZFP36 and ZFP36L1 to antigen affinity and PMA versus ionomycin stimulation. By selectively inhibiting TCR‐induced signalling pathways, we find that p38 MAPK, MEK1/2, and PKC contribute to inducing both ZFP36 and ZFP36L1 expression. By contrast, inhibition of calcineurin using cyclosporin A potently inhibits ZFP36L1 expression while increasing and prolonging ZFP36 expression. The *Zfp36* promoter contains many binding sites for the transcription factors ELK‐1/4 and few binding sites for NFAT, while the *Zfp36l1* promoter contains many NFAT binding sites and few ELK1/4 binding sites. Our findings suggest that the regulation of divergent transcription factors enables calcineurin to act as a signalling node that mediates the differential regulation of ZFP36 and ZFP36L1 during T cell activation.

## Graphical abstract

Proposed mechanism underlying the repression of ZFP36 and induction of ZFP36L1 by calcineurin. ELK‐1 is activated by stimuli and promotes the transcription of *Zfp36*. Calcineurin dephosphorylates active ELK‐1 to inhibit its positive effect on *Zfp36* transcription, while promoting NFAT‐mediated *Zfp36l1* transcription.

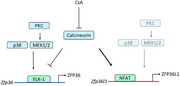

## 1 Introduction

T cell activation leads to proliferation and acquisition of effector functions, which are essential for the elimination of infections and ultimately the formation of memory. The T cell receptor is a key signal transducer which has been extensively characterised in terms of its proximal modes of signalling and downstream targets. One of the multiple pathways induced by TCR‐antigen engagement includes the bifurcating pathway that hydrolyses phosphatidylinositol 4,5‐bisphosphate into inositol trisphosphate and diacylglycerol. This leads to the activation of protein kinase C, the Ras‐ERK mitogen‐activated protein kinase (MAPK) pathways, and release of intracellular calcium (Ca^2+^) [1]. TCR stimulation also activates the p38 MAPK pathway and phosphoinositide 3‐kinases (PI3K), which orchestrate numerous downstream events to drive the proliferation and differentiation of the activated T cell [1].

Early events after T cell activation include the nuclear translocation of transcription factors (TFs) NF‐κB and NFAT that pre‐exist in the cytoplasm in an inactive state. An additional class of targets regulated early after T cell activation are RNA Binding Proteins (RBPs), which profoundly influence gene expression in the immune system [2, 3]. The regulatory activity of TFs and RBPs drives a wave of rapid “immediate early” gene transcription and “delayed early” genes for which gene transcription is independent of *de novo* protein synthesis [4]. Together, these pathways promote the transcription of secondary response genes—defined as genes which require *de novo* protein synthesis to be efficiently transcribed. Of TCR downstream targets, TFs and epigenetic regulators have been explored in the most detail, but some RBPs are also rapidly responsive to TCR stimulation, and these include ZFP36, which is an immediate early gene in non‐lymphoid cells [5].

The ZFP36‐family of RBP, three of which (ZFP36, ZFP36L1 and ZFP36L2) can be expressed by T cells, contains a tandem zinc finger RNA binding domain which interacts with AU‐rich sequences in RNAs with high affinity to direct the localisation and translation or degradation of the bound RNA [2, 6]. These RBPs have important roles in T cell function. They were initially shown to be repressors of cytokine production by T cells [7, 8, 9, 10, 11, 12]. Subsequently, evidence has emerged that these RBPs regulate many mRNAs in T cells and that these play a role in regulating the speed of T cell differentiation, the potency of cytotoxic T cells [13, 14] and the responsiveness of CD8^+^ T cells to IL‐2 [15].

ZFP36 and ZFP36L1 are rapidly and transiently induced following TCR engagement [13, 15], unlike ZFP36L2, which is expressed in resting T cells and induced slowly following TCR stimulation [16]. The overlapping expression patterns of ZFP36 and ZFP36L1 are suggestive of functional redundancy, and experiments using T cells lacking combinations of *Zfp36* and *Zfp36l1* have demonstrated redundancy [13, 14, 17, 18]. However, these and other studies [15, 19, 20] have also provided evidence for unique non‐redundant roles, in particular, in the context of differentiation and effector function. ZFP36L1 but not ZFP36 induction was shown to be highly sensitive to TCR affinity and critical for clonal selection of high‐affinity T cells [15]. How the expression of these RBPs is controlled by TCR signalling has not been investigated.

In addition to being transcribed from an immediate early gene, ZFP36 is also phosphorylated by MAP kinase‐activated protein kinase 2 (MK2) downstream of RAS‐MEK, and the p38 MAPK pathway [21, 22]. In a mouse macrophage cell line, the p38 MAPK and ERK pathways synergistically induce ZFP36 protein, but not mRNA [23]. The phosphorylation of ZFP36 has been shown to promote its stability, while simultaneously inhibiting its ability to promote RNA decay. Regulation of ZFP36 by MAPK‐induced phosphorylation has been shown to play a major role in modulating expression of *Tnf* and other pro‐inflammatory mRNAs in macrophages [24, 25, 26]. MK2 also phosphorylates ZFP36L1 [27], as does the PI3K‐activated protein kinase B/AKT [28] and ERK‐activated p90 ribosomal S6 kinase [29]. These post‐translational events also promote the accumulation of ZFP36L1 protein [30]. It is unknown whether and how these pathways influence the expression of ZFP36 family members in activated T cells.

Here, using *in vitro* expanded antigen‐experienced CD8^+^ T cells, we investigate the pathways that regulate ZFP36 and ZFP36L1 expression following TCR engagement. We identify calcineurin to be a point of divergent regulation between ZFP36 and ZFP36L1. We provide evidence that ZFP36L1 is principally an NFAT‐responsive gene, while ZFP36 is principally responsive to the ERK‐ELK transcription factor axis. We suggest that downstream of the TCR, calcineurin switches off expression of the immediate early gene ZFP36 and induces the more sustained expression of ZFP36L1.

## 2 Results

### 2.1 TCR Stimulation Induces Early and Transient ZFP36, but Sustained ZFP36L1 Expression

**FIGURE 1:**
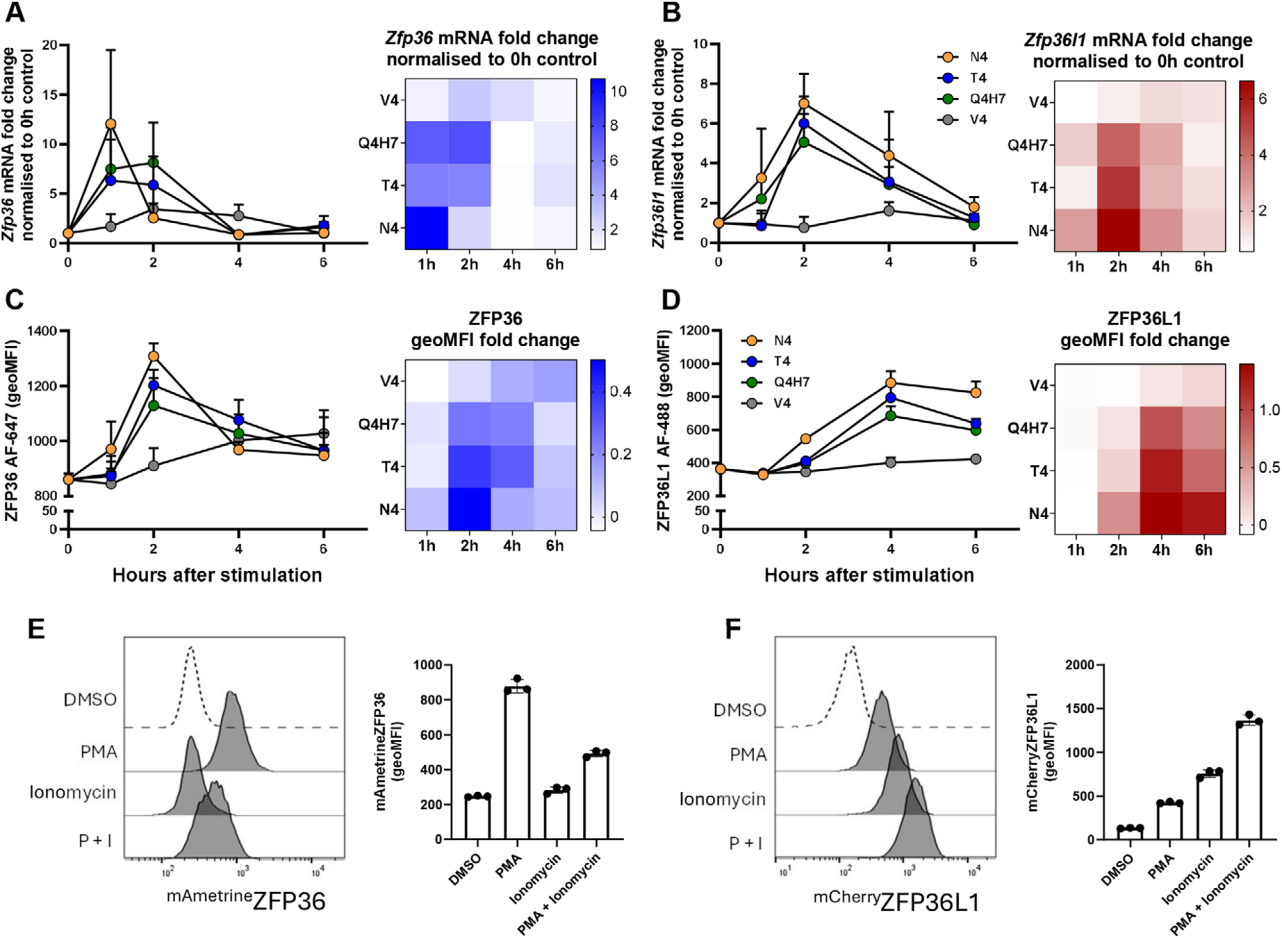
ZFP36 and ZFP36L1 expression exhibit distinct kinetics, sensitivity to antigen affinity and PKC versus Ca^2+^ signalling. Graphs show (A, B) *Zfp36* and *Zfp36l1* mRNA fold change, and (C, D) ZFP36 and ZFP36L1 geometric mean fluorescence intensity (geoMFI) in WT memory‐like T cells stimulated for up to 6 h with 0.1 nM N4, or lower‐affinity altered peptide ligands (T4, Q4H7, or V4), relative to unstimulated cells. mRNA fold change was quantified by the ∆∆Ct qPCR method, and proteins were detected by intracellular flow cytometry. (A–D) Heat maps summarise fold change in (A, B) *Zfp36* and *Zfp36l1* mRNA or (C, D) ZFP36 and ZFP36L1 protein abundance. Error bars show standard deviation of the mean (SD) of three biological replicates analysed in one experiment. (E) Detection of ^mAmetrine^ZFP36 and (F) ^mCherry^ZFP36L1 in memory‐like T cells stimulated for 3 h with 10 ng/mL PMA and/or 1 µM ionomycin or treated with DMSO alone. Representative histograms show ^mAmetrine^ZFP36 or ^mCherry^ZFP36L1 expression from one of three homozygous biological replicates. The geoMFI was quantified and plotted; error bars show the SD of three biological replicates analysed in one experiment.

Previous studies of ZFP36‐family member expression in terminally differentiated effector CD8^+^ T cells showed that ZFP36 and ZFP36L1 proteins were both transiently induced after TCR stimulation within a 24 h window [13]. To study this in non‐terminally differentiated cells, we expanded naïve OT1 CD8^+^ T cells in vitro to generate large numbers of cells with a memory‐like phenotype, as previously described [31], which could be used for flow cytometry, Western blotting and qPCR analysis following stimulation with peptide ligands which have a range of affinities for the OT1 TCR [32]. In these cells, TCR stimulation using the highest affinity N4 peptide caused rapid induction of *Zfp36* mRNA, which peaked by 1 h and decreased by 2 h after stimulation (Figure 1A). TCR agonists of lower affinity also rapidly induced *Zfp36* mRNA, but this remained higher at 2 h than in cells stimulated with N4 peptide and declined thereafter. N4 only induced greater *Zfp36* mRNA than lower‐affinity antigens 1 h after stimulation; at later timepoints, *Zfp36* mRNA was not proportional to antigen affinity. In comparison, *Zfp36l1* mRNA was highest 2 h after stimulation; at its peak, the fold change in mRNA was greatest with the highest affinity peptide and decreased according to peptide affinity (N4 > T4> Q4H7 > V4) (Figure 1B). At later timepoints, fold change of the *Zfp36l1* mRNA induced by N4 continued to be greater compared with the lower affinity peptides. The kinetics of *Zfp36l1* mRNA expression were consistent irrespective of peptide affinity, except for V4, which very weakly induced *Zfp36l1*. Thus, both transcripts are induced at the level of mRNA following TCR stimulation but with different kinetics and responses to antigen affinity.

We monitored the kinetics of protein expression at single‐cell resolution, in the same cells from which we harvested mRNA, by intracellular flow cytometry using antibodies specific for ZFP36 and ZFP36L1. The peak expression of ZFP36 was delayed compared with the peak expression of its transcript (Figure 1C). At this peak, but not thereafter, the amounts of ZFP36 scaled according to antigen affinity. ZFP36L1 also peaked later than its transcript and, at all timepoints analysed, the fold change of protein expression scaled according to peptide affinity (Figure 1D). The differing kinetics of ZFP36 and ZFP36L1 expression were also reflected in the frequencies of ZFP36^+^ and ZFP36L1^+^ cells (Figure S1); N4 stimulation induced the greatest proportion of cells expressing ZFP36 2 h post‐stimulation. The frequency of ZFP36L1^+^ cells was consistently higher versus lower affinity peptides at all timepoints analysed. The data show that ZFP36 displays an expression pattern in memory‐like cells that is consistent with it being an immediate early gene, while ZFP36L1 behaves as a delayed primary response gene [4] with expression linked to antigen affinity.

### 2.2 ZFP36 and ZFP36L1 Are Distinctly Sensitive to MAPK, PKC and Ca^2+^ Signalling

To identify the mechanisms regulating ZFP36 and ZFP36L1 expression, we used memory‐like cells derived from genetically modified mice in which the *Zfp36* and *Zfp36l1* loci were modified by insertion of open reading frames encoding the fluorescent proteins mAmetrine or mCherry in the same reading frame as the start codon of ZFP36 or ZFP36L1, respectively [15]. These alleles retain the transcriptional and post‐transcriptional regulatory elements of the endogenous transcripts, with the ^mAmetrine^ZFP36 and ^mCherry^ZFP36L1 reporter proteins predicted to be subject to the same post‐translational regulation as the unmodified proteins. We compared reporter protein expression in memory‐like T cells derived from ^mAmetrine^ZFP36 and ^mCherry^ZFP36L1 heterozygous or homozygous mice to validate that analysis of homozygous cells resulted in the greatest dynamic range of detection (Figure S2). We therefore used homozygous mice for all analysis using ^mAmetrine^ZFP36 and ^mCherry^ZFP36L1 reporter proteins, unless stated otherwise.

We stimulated memory‐like T cells with phorbol 12‐myristate 13‐acetate (PMA) and ionomycin, which mimic the bifurcating signals of TCR signal transduction via Phospholipase C. PMA induces signalling via PKC and Ras pathways in T cells, while ionomycin leads to Ca^2+^ release and the activation of calcineurin. Three hours post‐stimulation, PMA strongly induced ^mAmetrine^ZFP36 expression, while ionomycin alone stimulated very limited expression. Notably, ^mAmetrine^ZFP36 expression in response to combined PMA and ionomycin stimulation was less than that following stimulation with PMA alone (Figure 1E). In contrast, ^mCherry^ZFP36L1 expression was more strongly induced by ionomycin alone than by PMA alone, and these agents induced the greatest amounts of ^mCherry^ZFP36L1 when used together (Figure 1F). We confirmed these observations at additional timepoints using Western blotting and found preferential induction of ZFP36 by PMA (Figure S3A) and of ZFP36L1 by ionomycin (Figure S3B) up to 4 h post‐stimulation. After 1 h, ZFP36 was strongly induced by PMA and decreased after 2 h of stimulation. Ionomycin stimulation failed to induce ZFP36 expression that was detectable using our conditions for Western blotting. When added together with PMA, ionomycin slightly reduced ZFP36 expression (Figure S3A). Thus, ZFP36 and ZFP36L1 respond differently to PMA and ionomycin.

**FIGURE 2:**
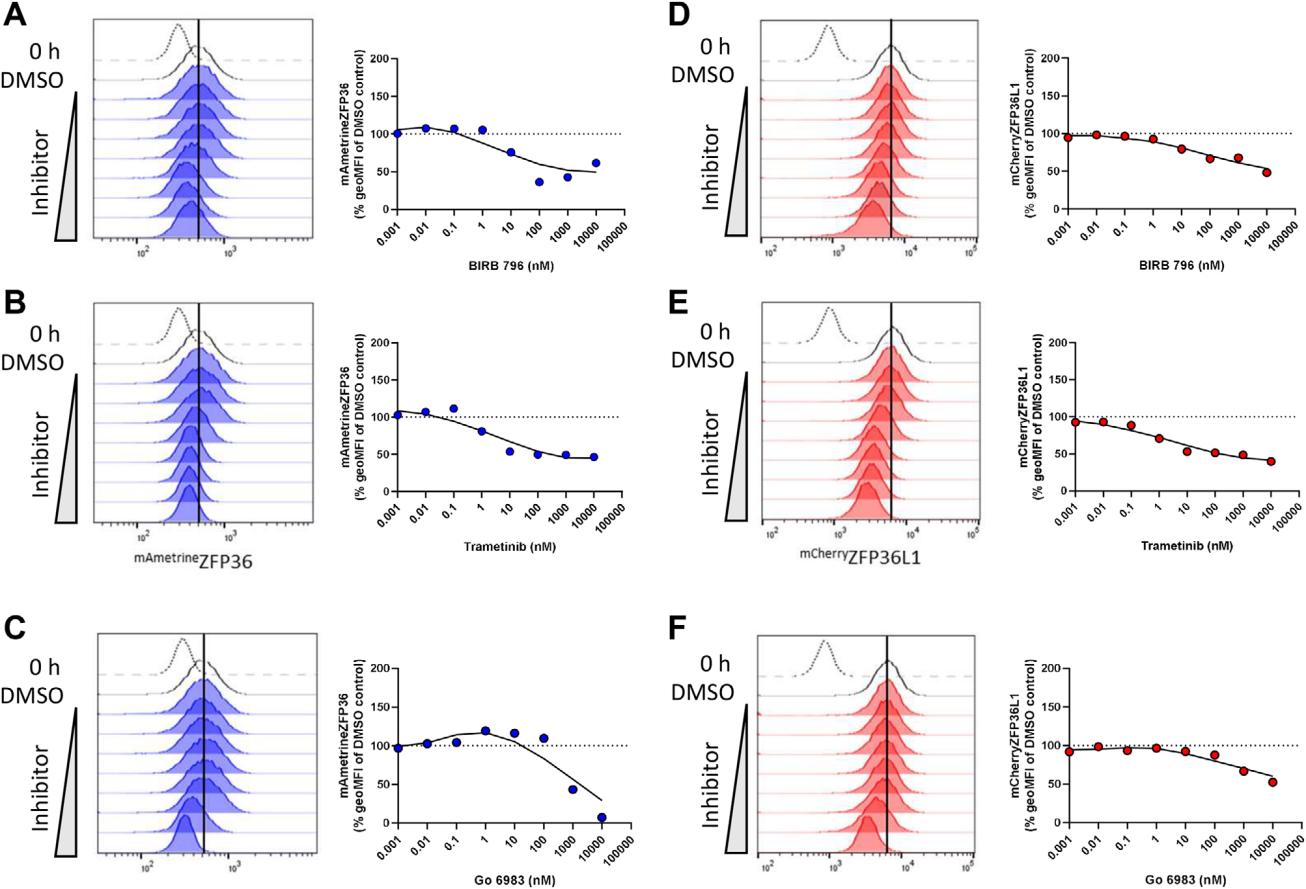
p38 MAPK, MEK1/2, and PKC signalling are common pathways that promote the expression of both ZFP36 and ZFP36L1. Memory‐like T cells were pre‐treated for 30 min with 0.001, 0.01, 0.1, 1, 10, 100, 1000, or 10,000 nM BIRB 796 (p38 MAPK inhibitor), Go6983 (PKC inhibitor), or Trametinib (MEK1/2 inhibitor) or DMSO before being stimulated for 4 h with 0.1 nM N4 in the presence of each inhibitor. Histograms show (A–C) ^mAmetrine^ZFP36 geoMFI or (D–F) ^mCherry^ZFP36L1 expression after 4 h of stimulation. The broken line shows 0 h unstimulated control, and the unfilled histogram shows stimulated DMSO‐only control. Filled histogram shows ^mAmetrine^ZFP36 or mCherryZFP36L1 geoMFI of cells stimulated in the presence of the indicated inhibitor. The effect of each inhibitor on ^mAmetrine^ZFP36 or ^mCherry^ZFP36L geoMFI was analysed by normalising the geoMFI following inhibitor treatment as a percentage of the DMSO‐treated stimulated control. The ^mAmetrine^ZFP36 or ^mCherry^ZFP36L1 geoMFI of resting cells was subtracted as background fluorescence before calculating the percentage inhibition of expression. Data shown are from one biological replicate and are representative of a total of three ^mAmetrine^ZFP36 ^mCherry^ZFP36L1 homozygous biological replicates analysed in one experiment.

We further investigated the signalling pathways regulating the expression of ZFP36 and ZFP36L1 in the presence of small molecule inhibitors, which were added to memory‐like T cells prior to TCR stimulation. Four hours after stimulation, BIRB 796 (p38 MAPK inhibitor), trametinib (MEK1/2 inhibitor) and Go6983 (PKC inhibitor) each inhibited expression of ^mAmetrine^ZFP36 (Figure 2A–C). Inhibiting p38 MAPK or MEK1/2 pathways also inhibited ^mCherry^ZFP36L1 induction (Figure 2D,E). Inhibition of PKC signalling resulted in a modest inhibition of ^mCherry^ZFP36L1 expression (Figure 2F) compared with the effect of Go6983 on ^mAmetrine^ZFP36 expression. Taken together, these data indicate that ZFP36 and ZFP36L1 are both induced by p38 MAPK, MEK1/2 and PKC signalling. The inhibition of the upstream signalling node PKC dominantly regulates ZFP36 expression, but not ZFP36L1, suggesting non‐redundancy of this pathway for the expression of ZFP36. These pathways are likely to underlie the ability of PMA to induce both ZFP36 and ZFP36L1.

### 2.3 Calcineurin Differentially Regulates ZFP36 and ZFP36L1 Expression

**FIGURE 3:**
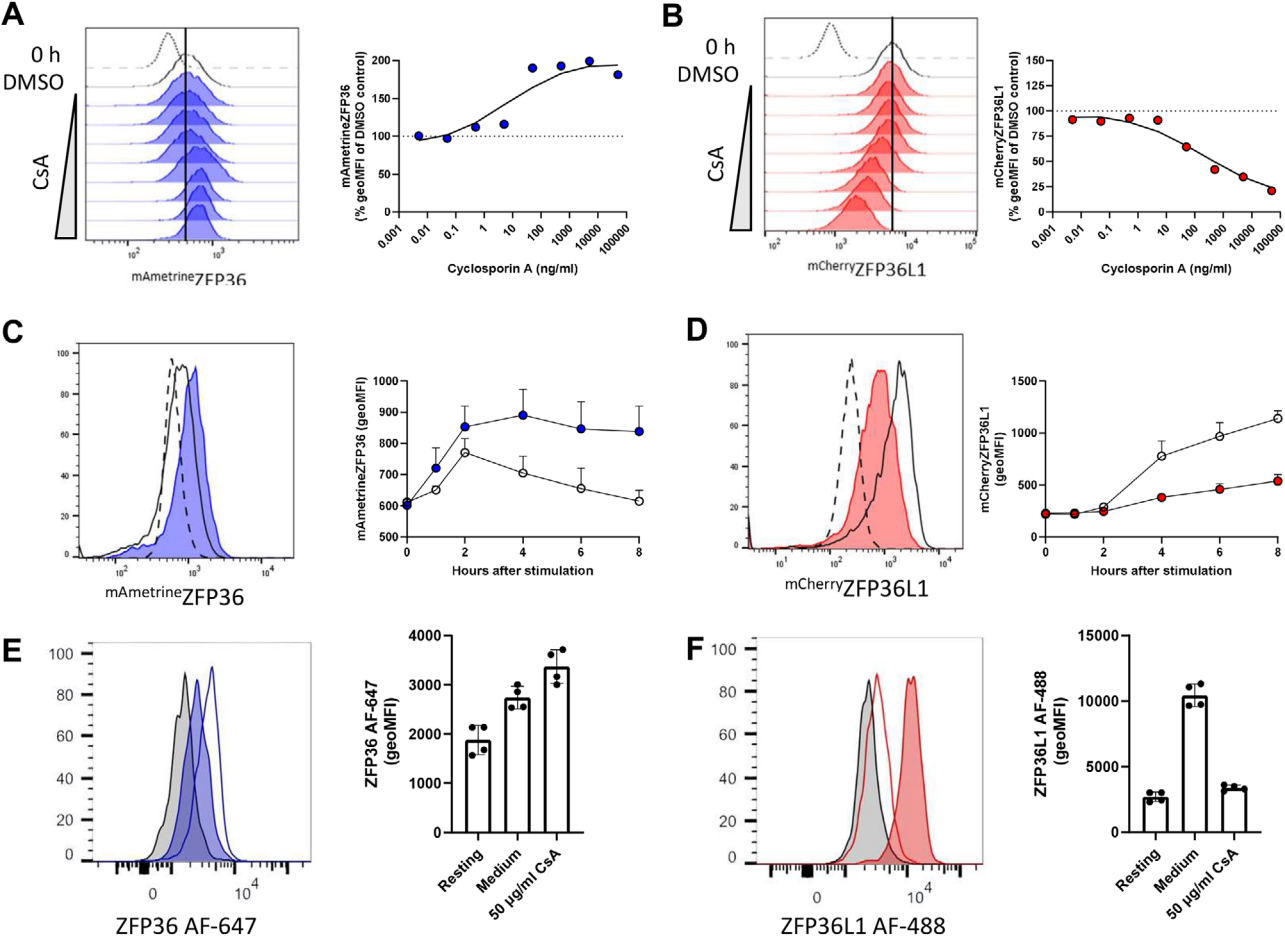
Cyclosporin A increases ZFP36 and inhibits ZFP36L1 expression. (A, B) Memory‐like T cells were pre‐treated for 30 min with 0.005, 0.05, 0.5, 5, 50, 500, 5000, or 50,000 ng/mL cyclosporin A (CsA) (calcineurin inhibitor) or DMSO control before being stimulated for 4 h with 0.1 nM N4 in the presence of CsA or DMSO. Histograms show ^mAmetrine^ZFP36 or ^mCherry^ZFP36L1 geoMFI after 4 h of stimulation. The broken line shows 0 h unstimulated control, and the unfilled histogram shows stimulated DMSO control. Filled histogram shows geoMFI of cells stimulated in the presence of CsA. For quantification, the effect of CsA was analysed by normalising the ^mAmetrine^ZFP36 or ^mCherry^ZFP36L1 geoMFI following inhibitor treatment as a percentage of the DMSO‐treated stimulated control. The ^mAmetrine^ZFP36 or ^mCherry^ZFP36L1 geoMFI of resting cells was subtracted as background fluorescence before calculating the percentage inhibition of expression. Data shown are from one biological replicate and are representative of a total of three homozygous biological replicates analysed in one experiment. (C, D) Memory‐like T cells were pre‐treated for 30 min with 500 ng/mL CsA before being stimulated for up to 8 h with 0.1 nM N4 in the presence of CsA or DMSO. Representative histograms show ^mAmetrine^ZFP36 and ^mCherry^ZFP36L1 expression from one of three homozygous biological replicates. The broken line shows 0 h unstimulated control, and the unfilled histogram shows stimulated DMSO control. Filled histogram shows geoMFI of cells stimulated in the presence of CsA. The geoMFI was quantified and plotted; error bars show the standard deviation of the mean (SD) of three biological replicates analysed in one experiment. (E, F) *Ex vivo* memory T cells were stimulated for 3 h with 10 ng/mL PMA and 1 µM ionomycin in the presence or absence of CsA. Representative histograms show (E) ZFP36 or (F) ZFP36L1 expression in unstimulated (light grey), or PMA/ionomycin stimulated memory cells in the absence (filled coloured histogram) and presence (open histogram) of CsA in one of two biological replicates. ZFP36 and ZFP36L1 geoMFI were quantified, and the geoMFI of the two biological replicates analysed in one experiment were plotted, showing two technical replicates from each biological replicate. Error bars show the standard deviation of the mean (SD).

While PMA stimulation alone was sufficient to induce both ZFP36 and ZFP36L1 expression (Figure 1E,F), ZFP36 and ZFP36L1 exhibited contrasting sensitivity to ionomycin. We therefore further investigated the role of TCR‐induced Ca^2+^ signalling in regulating ZFP36 and ZFP36L1 expression through the use of the calcineurin inhibitor cyclosporin A (CsA). Strikingly, ZFP36 expression 4 h post‐stimulation was higher in the presence of CsA than in cells treated with DMSO (Figure 3A). By contrast, CsA was a potent dose‐dependent inhibitor of ^mCherry^ZFP36L1 induction by TCR stimulation (Figure 3B). CsA also increased ^mAmetrine^ZFP36 expression at 6‐ and 8 h after stimulation; this effect was increasingly pronounced as, in the absence of CsA, ^mAmetrine^ZFP36 expression was declining (Figure 3C). As observed at 4 h, the inhibition of ZFP36L1 expression by CsA was consistent at 6‐ and 8 h post‐stimulation (Figure 3D). Thus, CsA inhibits ^mCherry^ZFP36L1 expression but sustains the expression of ZFP36.

To extend our observations, we analysed memory‐like cells from mice expressing ^mAmetrine^ZFP36 and ^mCherry^ZFP36L1 by Western blotting in parallel with flow cytometry. N4 stimulation induced expression of ^mAmetrine^ZFP36 2 h post‐stimulation, which declined and was undetectable after 8 h (Figure S3C). In the presence of CsA, ^mAmetrine^ZFP36 expression was greater after 2 h of stimulation compared with untreated cells. ^mAmetrine^ZFP36 expression was also prolonged by the presence of CsA, as increased ^mAmetrine^ZFP36 expression was maintained up to 8 h post‐stimulation (Figure S3C). CsA inhibited N4‐induced ^mCherry^ZFP36L1 expression most potently 2–4 h after stimulation and notably reduced the accumulation of protein resolving at a lower molecular weight (Figure S3C), which may represent hypo‐phosphorylated ^mCherry^ZFP36L1.

To confirm the effects of CsA on the endogenous ZFP36 and ZFP36L1 proteins, we stimulated memory‐like cells derived from wild‐type OT1 mice *in vitro*. CsA inhibited ZFP36L1 and prolonged the expression of ZFP36 (Figure S3D). We also investigated whether CsA similarly regulated ZFP36 and ZFP36L1 expression in *ex vivo* memory T cells. CsA treatment increased ZFP36 expression (Figure 3E) while potently inhibiting ZFP36L1 expression to levels similar to those of resting cells (Figure 3F). The effects of CsA suggest that Ca^2+^ signalling via calcineurin phosphatase is a key pathway that mediates the differential regulation of ZFP36 and ZFP36L1.

### 2.4 Calcineurin Regulates the Expression of *Zfp36* and *Zfp36l1* mRNAs

**FIGURE 4:**
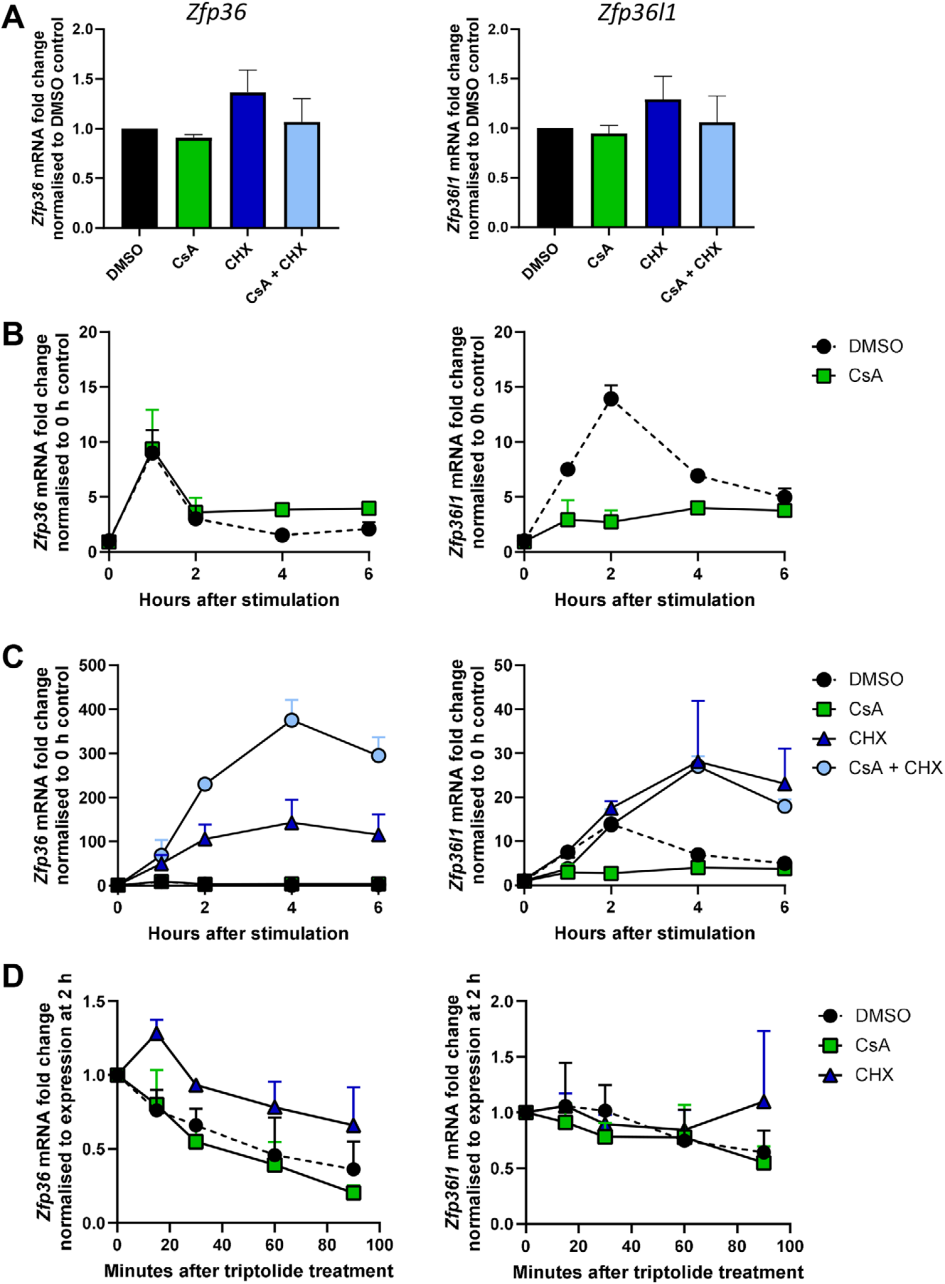
CsA prolongs *Zfp36* mRNA expression independently of *de novo* protein translation or transcript stability. (A) Abundance of *Zfp36* and *Zfp36l1* mRNA was measured in resting WT OT1 memory‐like T cells that were pre‐treated for 30 min with 500 ng/mL cyclosporin A (CsA) (calcineurin inhibitor), 10 µg/mL cycloheximide (CHX) (translation inhibitor) or DMSO control, analysed by ∆∆Ct qPCR analysis. (B–D) WT OT1 memory‐like T cells were stimulated for up to 6 h with 0.1 nM N4 in the presence of 500 ng/mL CsA, 10 µg/mL CHX or DMSO control as indicated. (B, C) The abundance of *Zfp36* and *Zfp36l1* mRNA was determined by ∆∆Ct qPCR analysis using cells pre‐treated with DMSO as a universal control. (D) After 2 h of stimulation, the cells were treated with 1 µM triptolide (transcription inhibitor) for up to 90 min. Fold‐change of *Zfp36* and *Zfp36l1* mRNA abundance was normalised to the abundance in each condition 2 h after stimulation, prior to triptolide treatment, using ∆∆Ct qPCR analysis. (A–D) Error bars show standard deviation of the mean of three biological replicates analysed in one experiment.

Both the rate of transcription and transcript stability determine the overall abundance of mRNA. To gain a mechanistic understanding of how CsA differentially regulates ZFP36 and ZFP36L1 expression, we investigated the effect of CsA on *Zfp36* and *Zfp36l1* mRNA abundance using qPCR analysis. The expression of *Zfp36* and *Zfp36l1* in resting cells was unaffected by CsA (Figure 4A). Following peptide stimulation, CsA treatment extended the duration of *Zfp36* mRNA expression, which remained elevated at 4‐ and 6 h post‐stimulation without increasing the peak abundance 2 h after stimulation. By contrast, CsA limited the induction of *Zfp36l1* mRNA to a maximum of four‐fold versus 14‐fold in DMSO‐treated control cells (Figure 4B).

ZFP36 protein expression is subject to strong post‐transcriptional regulation, which includes its own autoregulation [33]. Deletion of AU‐rich elements in the 3′UTR of *Zfp36* resulted in increased ZFP36 protein and greater resilience of mice to inflammation‐induced disease [34]. The protein synthesis inhibitor cycloheximide (CHX) is thought to enhance *Zfp36* mRNA expression by inhibiting a negative feedback mechanism, mediated by *de novo* translated proteins, including ZFP36 itself, that promotes *Zfp36* mRNA decay (Figure S5) [33, 35]. CHX should abolish the effect of CsA if, upon T cell activation, CsA acts as an inhibitor of these negative regulators of *Zfp36* mRNA. To test whether negative feedback regulators of *Zfp36* mRNA are affected by CsA, we pre‐treated memory‐like cells with CHX. The abundance of both *Zfp36* and *Zfp36l* *1* mRNA was increased upon pretreatment with CHX between 2 and 6 h after stimulation. However, the addition of CsA in the presence of CHX further increased the expression of *Zfp36* mRNA but not *Zfp36l1* mRNA (Figure 4C). This suggests that induction of *Zfp36* mRNA expression by CsA is not via the inhibition of a negative feedback mechanism mediated by ZFP36 or other regulators of mRNA induced upon T cell activation.

We further investigated whether CsA modulates ZFP36 expression via stabilisation of *Zfp36* mRNA. Inhibition of transcription using triptolide (TRP), which causes rapid proteasomal degradation of RNA polymerase II, enables analysis of mRNA stability [36]. We analysed the stability of *Zfp36* or *Zfp36l1* mRNA between 2‐ and 4 h after stimulation, when the *Zfp36* transcript declined in DMSO‐treated control cells but not in the presence of CsA (Figure 4B). 90 minutes after TRP treatment, the abundance of *Zfp36* mRNA had rapidly declined by 64% in the DMSO‐treated control compared with 80% in the presence of CsA. In contrast, higher *Zfp36* mRNA abundance was maintained for longer following CHX treatment, which increased the half‐life of *Zfp36* mRNA to be greater than 90 min. Thus, CHX but not CsA treatment resulted in increased *Zfp36* mRNA stability. The stability of *Zfp36l1* mRNA was not affected by the presence of either CsA or CHX (Figure 4D). We therefore conclude that the induction of ZFP36 expression by CsA is not due to the post‐transcriptional stabilisation of the *Zfp36* transcript.

ZFP36 protein stability can be regulated by MAPK‐dependent and MAPK‐independent pathways [37, 38]. We, therefore, investigated whether the inhibition of calcineurin promotes the accumulation of ZFP36 protein. To analyse the effect of CsA on the stability of ZFP36 protein and its degradation, we used either cycloheximide or the proteasome inhibitor MG‐132 to inhibit *de novo* translation or block the degradation of ZFP36, respectively. We added these compounds after 2 h of stimulation with N4, when ^mAmetrine^ZFP36 expression is near its maximum. The addition of CHX had a limited effect on ^mAmetrine^ZFP36 compared with the DMSO control at 2–6 h after stimulation, indicating that the majority of ZFP36 protein had already been translated in the first 2 h of stimulation and that it was relatively stable (Figure S6A,B). MG‐132 treatment increased ^mAmetrine^ZFP36 expression at 4–6 h post‐stimulation, indicating that ZFP36 is subject to proteasomal degradation at these timepoints (Figure S6C). These data indicate there is little impact of CsA on ZFP36 protein stability. We conclude that the effects of CsA are mostly at the level of *Zfp36* mRNA transcription.

### 2.5 Calcineurin Sensitive Transcription Factors Can Explain the Differential Expression of ZFP36 and ZFP36L1 in Response to CsA

**FIGURE 5:**
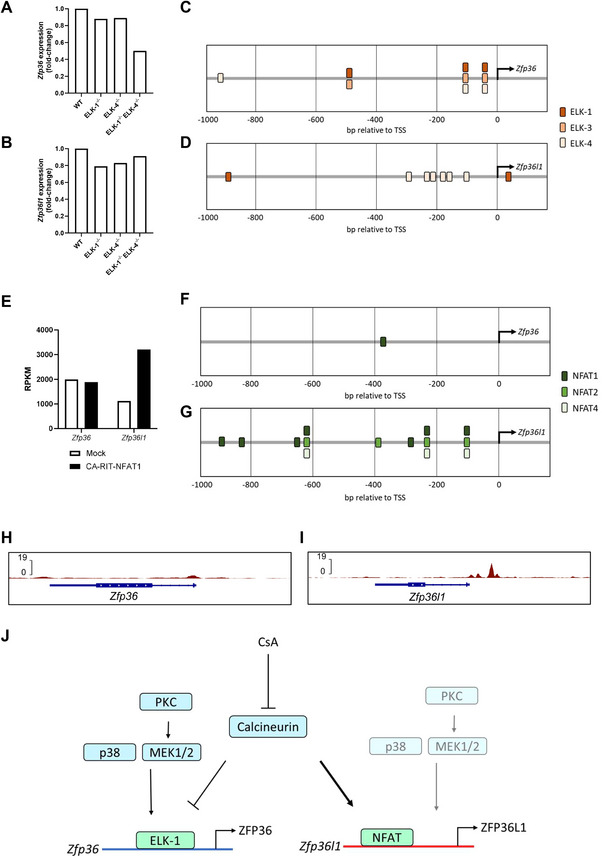
ELK and NFAT may underlie the divergent regulation of *Zfp36* and *Zfp36l1* by calcineurin. (A) *Zfp36* or (B) *Zfp36l1* microarray probe signal induced by 30 min plate‐bound CD3 stimulation in ELK mutant double‐positive thymocytes as a ratio compared with wildtype (WT). Data from Costello et al. [40]. (C, D) Graphical representation of ELK‐family transcription factor binding sites in the (C) *Zfp36* and (D) *Zfp36l1* promoters; analysis from the Eukaryotic Promoter Database [41] using a cut‐off p‐value of 0.001. (E) RPKM values from RNA‐Seq for *Zfp36* and *Zfp36l1* expression in CA‐RIT‐NFAT1‐transduced or mock‐transduced CD8^+^ T cells taken from Martinez et al. [46] (F, G) Graphical representation of NFAT‐family transcription factor binding sites in the (F) *Zfp36* and (G) *Zfp36l1* promoters as in panel (C, D). (H, I) ChIP‐seq data from Martinez et al. [46] for endogenous NFAT binding to (H) *Zfp36* or (I) *Zfp36l1* in WT cells stimulated with PMA and ionomycin for 1 h. (J) Visualisation of the proposed mechanisms underlying the negative regulation of ZFP36 and induction of ZFP36L1 by calcineurin. Activation of ELK‐1 by p38 MAPK, MEK1/2 and PKC signalling promotes the transcription of *Zfp36*. Calcineurin negatively regulates ELK‐1 to inhibit *Zfp36* transcription. In contrast, calcineurin promotes NFAT‐mediated *Zfp36l1* transcription and is the dominant pathway inducing ZFP36L1 expression compared with a lesser role of p38 MAPK, MEK1/2 and PKC. Inhibition of calcineurin by CsA results in increased ZFP36 and inhibits ZFP36L1 expression.

Next, we investigated if the opposing regulation of *Zfp36* and *Zfp36*l1 mRNA may be mediated by different transcription factors which regulate *Zfp36* and *Zfp36l1*. As MEK1/2 and p38 MAPK are key pathways regulating ZFP36 expression in memory‐like T cells (Figure 2A,B) we considered the transcription factor ELK‐1, a member of the ternary complex factors (TCFs) subfamily of transcription factors, which has been shown to promote *Zfp36* mRNA expression in MCF‐7 cells downstream of ERK1/2 activation in response to epidermal growth factor (EGF) stimulation [39]. In double positive thymocytes lacking ELK‐1 and ELK‐4, *Zfp36* expression was 0.5‐fold lower as a ratio compared with WT following plate‐bound anti‐CD3 stimulation [40] (Figure 5A). *Zfp36l1* mRNA showed only minor reduction in the absence of ELK‐1 and ELK‐4. Moreover, there was no additive effect on *Zfp36l1* mRNA in double‐deficient thymocytes (Figure 5B). Promoter analysis using the Eukaryotic Promoter Database [41] identified three putative binding sites for both ELK‐1 and ELK‐4 that were within a few hundred bases of the *Zfp36* transcription start site and thus could promote *Zfp36* transcription (Figure 5C). The *Zfp36l1* promoter region contains multiple ELK‐4 binding sites but just two ELK‐1 binding sites, one of which lies just downstream of the transcription start site (Figure 5D). The greater number and position of the ELK‐1 binding sites in the *Zfp36* promoter compared with the *Zfp36l1* promoter suggest that ELK‐1 may have a greater role in regulating *Zfp36* than *Zfp36l1* transcription. Notably, two independent studies demonstrated that ELK‐1 is dephosphorylated by calcineurin at phosphoserine 383 (S383) and that this inhibits its transcriptional activity [42, 43]. We therefore propose that the prolonged expression of *Zfp36* mRNA in the presence of CsA is, at least in part, due to loss of calcineurin‐mediated ELK‐1 inhibition.

Dephosphorylation of NFAT‐family transcription factors by calcineurin facilitates their nuclear translocation and transcriptional activity [44]. When NFAT interacts with Fos‐Jun (AP‐1), cooperative NFAT:AP‐1 complexes are formed, which have been shown to promote the induction of cytokines such as IL‐2 [45, 46]. To examine the effect of NFAT on *Zfp36* and *Zfp36l1*, we examined RNA‐seq data generated from CD8^+^ T cells expressing a constitutively active form of NFAT1, which cannot interact with AP‐1 [46]. In these cells, the constitutively active NFAT1 promotes increased expression of *Zfp36l1*, but *Zfp36* expression is unchanged (Figure 5E). Analysis of the promoter regions of *Zfp36 and Zfp36l1* identifies only a single NFAT1 binding site in the *Zfp36* gene (Figure 5F) compared with seven NFAT1, four NFAT2 and three NFAT4 binding sites in the *Zfp36l1* gene (Figure 5G). Furthermore, CHIP‐seq data for NFAT in T cells [46] show much greater NFAT binding to the *Zfp36l1* gene promoter than to the *Zfp36* gene (Figure 5H,I). The enrichment of NFAT binding sites in the *Zfp36l1* compared with the *Zfp36* gene is consistent with *Zfp36l1* being more responsive to ionomycin than *Zfp36*.

## 3 Discussion

Here, we provide evidence that calcineurin regulates ZFP36 and ZFP36L1 expression in CD8^+^ memory‐like T cells by enforcing the transient expression of ZFP36 while promoting sustained ZFP36L1 expression. Taken together, our data lead us to propose a model for the divergent regulation of ZFP36 and ZFP36L1 expression mediated by the calcineurin‐responsive ELK‐family and NFAT‐family transcription factors (Figure 5J). This provisional model will require additional validation, focusing on the regulation of transcription factors and the transcription of the *Zfp36* and *Zfp36l1* genes in activated T cells and exploring whether calcineurin inhibitors can prolong ZFP36 expression in non‐T cells.

Given that CsA is a potent immunosuppressant [47] and that both ZFP36 and ZFP36L1 have previously been shown to limit T cell‐mediated inflammatory and immune responses [13, 14, 17, 18], our observations are relevant to the mechanism of action of CsA as an immunosuppressant. The immunosuppressive mechanism of action of CsA is partly explained by the inhibition of NFAT‐mediated transcription required for T cell activation [44]. However, CsA was also reported to inhibit the cytokines TNF‐β and Lymphotoxin‐β independently of an effect on NFAT [48]. By inhibiting the dephosphorylation of ELK1, CsA prolongs the expression of *Zfp36* mRNA, enabling the protein to be expressed for an extended period. Interestingly, CsA has also been reported to inhibit the activation of p38 MAPK [49, 50]. In this scenario, when p38/MK2‐mediated phosphorylation of ZFP36 is diminished, the RBP is better able to promote mRNA decay. Thus, CsA may extend the expression and function of ZFP36 beyond its narrow duration as an immediate early gene to limit T cell activation. This suggestion is consistent with the observation that mice with prolonged ZFP36 expression, resulting from removal of the autoinhibitory sequence in the *Zfp36* 3′UTR, show much reduced disease severity in an experimental autoimmune encephalitis, a T cell‐driven autoimmune disease [34].

Expression of ZFP36L1 in response to ionomycin stimulation alone, and in response to the constitutively active, AP‐1 independent, NFAT mutant, indicates that NFAT activation alone is sufficient for ZFP36L1 expression. The extent to which ZFP36L1 is part of the programme of T cell anergy/tolerance or exhaustion is interesting to consider, as ZFP36L1 plays a role in promoting the response to IL‐2 and limiting effector cell differentiation [15] and post‐transcriptional silencing of cytokine mRNA has been linked to the phenotype of anergic cells [51], but the underlying mechanisms remain uncharacterized.

In summary, the distinct regulation of ZFP36 and ZFP36L1 early after T cell activation suggests they play unique roles in shaping the dynamic transcriptome of the activated T cell.

## 4 Materials and Methods

### 4.1 Mice

Mice with modified *Zfp36* and *Zfp36l1* alleles acting as protein expression reporters have been previously described: Zfp36^2.1Tnr^ (MGI:7711587); (MGI:7325470) [15], with the OT1 TCR‐transgene (Vα2 and Vβ5 recognizing peptide residues 257‐264 of chicken ovalbumin in the context of H‐2K^b^) (MGI:3054907) and CD4cre transgene (MGI:2386448) were maintained on the C57BL/6 background at the Babraham Institute.

All mouse experimentation was approved by the Babraham Institute Animal Welfare and Ethical Review Body. Animal husbandry and experimentation complied with existing European Union and United Kingdom Home Office legislation and local standards under project licence PP9973990. Mice were bred and maintained in the Babraham Institute Biological Support Unit. Since the opening of this barrier facility (2009), no primary pathogens or additional agents listed in the FELASA recommendations have been confirmed during health monitoring surveys of the stock holding rooms. Ambient temperature was ∼19–21°C, and relative humidity was 52%. Lighting was provided on a 1 h light: 12 h dark cycle, including 15 min “dawn” and “dusk” periods of subdued lighting. After weaning, mice were transferred to individually ventilated cages with 1–5 mice per cage. Mice were fed CRM (P) VP diet (Special Diet Services) ad libitum and received seeds (e.g., sunflower, millet) at the time of cage‐cleaning as part of their environmental enrichment. Male and female mice were used in the experiments described here; all mice were aged between 6 and 20 weeks of age.

### 4.2 Antibodies and Flow Cytometry

For cell surface staining, single cell suspensions from cultured cells were prepared in FACS buffer (DPBS + 2 % FBS + 2 mM EDTA). All cells were blocked with Fc blocking antibody (24G2, BioXcell) and incubated with fixable cell viability dye eF780 (Thermo Fisher or BD) for 30 min at 4°C.

For intracellular staining of ZFP36 and ZFP36L1, cells were surface‐stained before being fixed with Cytofix/Cytoperm Fixation/Permeabilization Kit (BD) for 30 min at 4°C. Intracellular staining was performed in Permwash (BD) containing the intracellular antibody cocktail overnight at 4°C. The following antibody clones were used in the flow cytometry experiments: CD8 (53‐6.7), CD44 (IM7), CD69 (H1.2F3), CD62L (MEL‐14), ZFP36 (D1I3T), and ZFP36L1 (E6L6S). Data were acquired using a ZE5 Cell Analyzer (Bio‐Rad) or a Fortessa (Beckton Dickinson) flow cytometer equipped with 355, 405, 488, 561, and 640 nm lasers. Flow Cytometry data were analysed using FlowJo 10.8.1 software.

### 4.3 In Vitro Culture and Stimulation of Memory‐Like T Cells

Single cell suspensions of cells from the spleen and lymph nodes (inguinal, axillary and brachial) of mice were prepared by mechanical disruption of harvested tissues against a 40 µM cell strainer. Tissue harvesting and preparation of single cell suspensions were performed using RPMI‐1640 medium (Thermo Scientific) supplemented with 2% FBS (Thermo Scientific) and 2 mM EDTA (Thermo Scientific). Selective lysis of red blood cells using ACK lysis buffer (Thermo Scientific) was performed to enrich for leukocytes.

The engineered SAMBOK (also referred to as MEC.B7.SigOVA) mouse embryonic fibroblast (MEF) cell line, which expresses OVA_257–264_ presented by H‐2K^b^ and the co‐stimulatory ligand CD80, were used as APCs for the generation of memory‐like T cells as previously described [52]. SAMBOK cells were maintained in RPMI‐1640 media (Thermo Scientific) supplemented with 10% FBS and passaged by 1:15 dilution twice per week from a confluent density of 10 × 10^6^ cells/T75 flask by trypsinisation (Gibco TrypLE) for 5 min at 37°C.

The day before T cell activation (Day (D)‐1), 0.5 × 10^6^ SAMBOK cells were seeded per well in a 12‐well plate. The following day (D.0), the SAMBOK cells were washed twice with PBS (Sarstedt) to remove loosely adhered cells. Isolated leukocytes were seeded at a density of 2 × 10^6^ cells/mL in a total volume of 2 mL per well of a 12‐well plate. Cultures were maintained in Iscove's modified Dulbecco's medium (IMDM) (Thermo Scientific) medium supplemented with 10% FBS, 50 µM 2‐mercaptoethanol (Thermo Scientific), 100 U/mL penicillin, and 100 µg/mL streptomycin (Thermo Scientific). The cells were cultured at 37°C, 5% CO_2_. After 24 h (D.1), the cells in each well were gently resuspended and removed from the SAMBOK feeder layer before being combined in a 50 mL conical tube. The cells were centrifuged at 300*g* for 5 min at room temperature. The cells were seeded at a density of 1 × 10^6^ cells/mL in IMDM media containing 10 ng/mL recombinant murine IL‐7 (PeproTech or STEMCELL) in a 24‐well plate containing 2 mL/well. Three days post‐activation (D.3), the cells were reseeded in fresh IL‐7‐containing IMDM media at 1 × 10^6^ cells/mL in a final volume of 2 mL per well. From the fourth day of culture (D.4), 1 mL of the supernatant was discarded and replaced with 1 mL of fresh IMDM containing a 2× concentration of 20 ng/mL IL‐7. All in vitro assays were performed on memory‐like T cells between days 7–10 of culture. For all cell counting, cell number, diameter and percentage viability were quantified using a CASY Model TT cell counter (Scharfe systems/Roche). 10 µL of cell suspension was added to 10 mL of CASY ton. Cells between 7.5 and 13 µm in diameter were measured.

Memory‐like CD8^+^ T cells were harvested and washed with pre‐warmed supplemented IMDM and counted as previously described. For analysis by flow cytometry, cells were seeded at a final density of 1 × 10^6^ cells/mL in 96‐well U‐bottom plates. For western blot or qPCR analyses, cells were seeded at a final density of 1 × 10^6^–4 × 10^6^ cells/mL in 24‐well plates. For all assays, IL‐7 was used at a final concentration of 10 ng/mL. For analysis of antigen responses, memory‐like T cells were stimulated with N4 OVA peptide (Genscript) or the lower‐affinity variant OVA peptides T4, Q4H7, or V4. Alternatively, memory‐like T cells were stimulated with PMA or ionomycin. Stimulation mixes were prepared as 2× solutions, which were diluted 1:2 upon addition to the plated cells.

Inhibitor dilutions were first prepared as 1000× stocks by performing eight‐point 1:10 serial dilutions of each inhibitor in DMSO (Sigma). For the preparation of 2× inhibitor solutions, a 1:500 further dilution was performed of each inhibitor in IMDM media. This method maintained a constant concentration of DMSO across the range of inhibitor concentrations. To facilitate the necessary series of dilutions, cells were initially plated at 4× the final desired density with 40 ng/mL IL‐7 and rested for 30 min at 37°C and 5% CO_2_. The cells were pre‐treated for 30 min with a 2× stock of the relevant inhibitor or DMSO. Activation solutions containing a 1× inhibitor concentration and 2× N4 concentration were prepared by combining equal volumes of the appropriate 2× inhibitor solution (or DMSO) and 4 x N4 IMDM solution (or IMDM media alone). An equal volume of the activation solutions was added to the pre‐treated cells in culture and returned to the incubator. The inhibitors used were BIRB 796 (Insight Biotechnology), CsA (Sigma‐Aldrich), Go 698 (inhibits PKCα, PKCβ, PKCγ, PKCδ, and PKCζ) (Insight Biotechnology), and Trametinib (Insight Biotechnology).

### 4.4 Analysis of the Effect of CsA on *Ex Vivo* Memory T Cells

Splenocytes were isolated from 20‐week‐old WT mice according to the method described above to generate single‐cell suspensions. The splenocytes were pre‐treated with 50 µg/mL CsA for 30 min before stimulation with 10 ng/mL PMA and 1 µM ionomycin. For flow cytometry analysis of ZFP36 and ZFP36L1, CD8^+^ memory T cells were gated according to the strategy described in Figure S4. The experiment was performed with two biological replicates, with two technical replicates respectively.

### 4.5 Inhibition of mRNA Transcription, Protein Translation or Proteasomal Degradation

To analyse mRNA stability, memory‐like T cells were pre‐treated with 500 ng/mL CsA or DMSO for 30 min. The cells were stimulated for 2 h with N4 peptide before 1 µM triptolide (CST) or DMSO was added at a final concentration of up to 90 min. To analyse superinduction of *Zfp36* and *Zfp36l1* mRNA, memory‐like T cells were pre‐treated for 30 min with 500 ng/mL CsA, 10 µg/mL cycloheximide (CHX) (Sigma‐Aldrich) or DMSO. The cells were then stimulated with N4 for up to 6 h. The cells were then harvested and frozen on dry ice for qPCR analysis.

To inhibit translation and enable analysis of protein stability, the cells were first pre‐treated for 30 min with 500 ng/mL CsA or DMSO. Memory‐like T cells were stimulated for 2 h before 10 µg/mL CHX or DMSO was added, and the cells were stimulated for a further 4 h. To inhibit proteasomal degradation, memory‐like T cells were stimulated for 2 h before the addition of 10 µM MG‐132 (Sigma‐Aldrich) or DMSO and the cells were stimulated for up to a further 4 h. The cells were harvested and prepared for flow cytometry analysis.

### 4.6 Western Blotting

Whole cell lysates were prepared using 1× RIPA buffer [SDS 0.1% (w/v), sodium deoxycholate 0.4% (w/v), NP‐40 0.5% (v/v), 100 mM NaCl, 50 mM Tris‐HCl, pH 7.4], supplemented with 1:100 protease inhibitor cocktail (Sigma), phosphatase inhibitor cocktail III (Sigma), and 2 U/mL Benzonase (Sigma). Protein lysate concentration was determined using the Pierce BCA protein assay kit (Thermo Scientific). Lysates were denatured for 5 min at 97°C with 4× Laemmli buffer (200 mM Tris‐HCl (pH 6.8), 8 % w/v SDS, 40% v/v glycerol, 0.08% w/v bromophenol blue) containing 5% β2‐mercaptoethanol. Protein lysate concentrations were normalised to enable the loading of an equivalent mass of protein per lane of a 10% polyacrylamide SDS‐PAGE gel. 5 µL of Precision Plus Protein Dual Colour Standard (Bio‐Rad) was loaded in one lane as a molecular weight marker. 1 µg of protein lysates of HEK293 cells expressing FLAG‐tagged mouse ZFP36 or ZFP36L1 was loaded as a positive control for antibody staining. The proteins were resolved by running at 120 V for ∼1.5 h before being transferred to a nitrocellulose membrane using the iBlot II transfer device (Thermo Scientific) at 15 V for 6 min. Ponceau staining was performed to visualise protein transfer before the membrane was washed with Milli‐Q water. The membrane was incubated in Intercept TBS blocking buffer (Li‐Cor), a ready‐to‐use blocker formulation, for 2 h at room temperature with constant shaking. Following blocking, the membrane was incubated with primary antibodies diluted in intercept buffer overnight at 4°C with constant shaking. The membrane was washed three times with TBS‐T (0.05% Tween‐20) and three times with TBS before being incubated with secondary antibodies diluted in intercept buffer for 2 h at room temperature. The membrane was washed again before image acquisition using the Odyssey CLx (Li‐Cor) and analysed using the ImageStudio Lite version 5.2 (Li‐Cor).

The membranes were probed using the following primary antibodies: ZFP36 (Origene 3D10), ZFP36L1 (CST BRF1/2 or E6L6S), GAPDH (CST D16H11), and α‐tubulin (CST DM1A). Secondary antibody staining was performed using antibodies anti‐mouse IgG IRDye800CM (Licor) and anti‐rabbit IgG IRDye680RD (Licor).

### 4.7 Real‐Time PCR Analysis

For the generation of samples for qPCR analysis, cells were thoroughly resuspended and harvested into at least twice the volume of ice‐cold PBS in 15 mL Falcon tubes. The cells were centrifuged at 300*g* for 5 min at 4°C before the supernatant was discarded. The cells were washed in PBS and transferred to a 1.5 mL Eppendorf tube before being centrifuged at 13,000*g* for 1 min at 4°C. The supernatant was removed, and the cell pellets were snap‐frozen on dry ice and stored at –70°C. RNA was extracted using the RNeasy Mini Kit (Qiagen) according to the manufacturer's instructions, including optional on‐column DNase treatment using the RNase‐Free DNase Set (Qiagen). RNA concentration was quantified using NanoDrop One (Nanodrop). RNA concentrations were normalised prior to cDNA synthesis using the RT RevertAid first‐strand cDNA synthesis kit (Thermo Scientific) according to the manufacturer's instructions. cDNA synthesis reactions were performed using the T100 Thermal Cycler (Bio‐Rad) using the following programme: 25°C for 5 min, 42°C for 60 min, and 70°C for 5 min. Quantitative real‐time PCR was performed using TaqMan universal PCR master mix (Thermo Scientific) and probes detailed below. Reactions were performed as technical triplicates in 384‐well plates with nuclease‐free water (Qiagen) used as a no‐template control (NTC). qPCR reactions were performed using the CFX384 Touch Real‐Time PCR Detection System (Bio‐Rad). The thermal cycling parameters were as follows: 50°C for 2 min, 95°C for 10 min, 40 cycles of 95°C for 15 s followed by 60°C for 1 min. The data were exported using the CFX Manager Software v. 3.1 (Bio‐Rad) and analysed in Excel v.2408 (Microsoft), where the data were analysed according to the ΔΔCt method using *Rpl32 (*Mm07306626_gH) as a housekeeping gene to normalise against. qPCR primers for *Zfp36 (*Mm00457144_m1) and *Zfp36l1* (Mm01304623_g1) and *Rpl32* were from Thermo Scientific.

### 4.8 Data Analysis

Data were analysed and visualised using GraphPad Prism version 10.

## Author Contributions

Marian Jones Evans: conceptualisation; methodology; investigation; validation; formal analysis; visualisation; writing original draft preparation. Twm J. Mitchell: conceptualisation; methodology; investigation. Georg Petkau: conceptualisation, investigation and writing. Magdalena Zaucha: conceptualisation; investigation; formal analysis. Martin Turner: conceptualisation, supervision, funding acquisition, writing review and editing.

## Conflicts of Interest

Some work on an unrelated project in M. T.’s lab is funded by AZ. The remaining authors declare no conflicts of interest.

## Supporting information




**Supporting file 1**: eji70085‐sup‐0001‐SuppMat.pdf

## Data Availability

The data that support the findings of this study are available from the corresponding author upon reasonable request.

## References

[1] A. H. Courtney , W.‐L. Lo , and A. Weiss , “TCR Signaling: Mechanisms of Initiation and Propagation,” Trends in Biochemical Sciences 43 (2018): 108–123, 10.1016/j.tibs.2017.11.008. 29269020 PMC5801066

[2] M. Fu and P. J. Blackshear , “RNA‐binding Proteins in Immune Regulation: A Focus on CCCH Zinc Finger Proteins,” Nature Reviews Immunology 17 (2016): 130–143, 10.1038/nri.2016.129. PMC5556700 27990022

[3] M. Turner and D. M. MD , “RNA‐binding Proteins Control Gene Expression and Cell Fate in the Immune System,” Nature Immunology 19 (2018): 120–129, 10.1038/s41590-017-0028-4. 29348497

[4] J. W. Tullai , M. E. Schaffer , S. Mullenbrock , G. Sholder , S. Kasif , and G. M. Cooper , “Immediate‐Early and Delayed Primary Response Genes Are Distinct in Function and Genomic Architecture*,” Journal of Biological Chemistry 282 (2007): 23981–23995, 10.1074/jbc.m702044200. 17575275 PMC2039722

[5] W. S. Lai , D. J. Stumpo , and P. J. Blackshear , “Rapid Insulin‐stimulated Accumulation of an mRNA Encoding a Proline‐rich Protein,” Journal of Biological Chemistry 265 (1990): 16556–16563, 10.1016/s0021-9258(17)46259-4. 2204625

[6] P. Kovarik , A. Bestehorn , and J. Fesselet , “Conceptual Advances in Control of Inflammation by the RNA‐Binding Protein Tristetraprolin,” Frontiers in immunology 12 (2021): 751313, 10.3389/fimmu.2021.751313. 34603339 PMC8484758

[7] R. L. Ogilvie , M. Abelson , H. H. Hau , I. Vlasova , P. J. Blackshear , and P. R. Bohjanen , “Tristetraprolin Down‐Regulates IL‐2 Gene Expression Through AU‐rich Element‐mediated mRNA Decay,” Journal of Immunology (Baltimore, Md: 1950) 174 (2005): 953–961. 15634918 10.4049/jimmunol.174.2.953

[8] R. L. Ogilvie , J. R. SternJohn , B. Rattenbacher , et al., “Tristetraprolin Mediates Interferon‐γ mRNA Decay*,” Journal of Biological Chemistry 284 (2009): 11216–11223, 10.1074/jbc.m901229200. 19258311 PMC2670126

[9] Q. Wang , H. Ning , H. Peng , et al., “Tristetraprolin Inhibits Macrophage IL‐27‐Induced Activation of Antitumour Cytotoxic T Cell Responses,” Nature Communications 8 (2017): 867, 10.1038/s41467-017-00892-y. PMC5636828 29021521

[10] M. J. Moore , N. E. Blachere , J. J. Fak , et al., “ZFP36 RNA‐Binding Proteins Restrain T Cell Activation and Anti‐Viral Immunity,” Elife 7 (2018): e33057, 10.7554/elife.33057. 29848443 PMC6033538

[11] F. Salerno , S. Engels , B. M. V. den , et al., “Translational Repression of Pre‐Formed Cytokine‐Encoding mRNA Prevents Chronic Activation of Memory T Cells,” Nature Immunology 19 (2018): 828–837, 10.1038/s41590-018-0155-6. 29988089 PMC6643272

[12] H. Peng , H. Ning , Q. Wang , et al., “Tristetraprolin Regulates TH17 Cell Function and Ameliorates DSS‐Induced Colitis in Mice,” Frontiers in Immunology 11 (2020): 1952, 10.3389/fimmu.2020.01952. 32922402 PMC7457025

[13] G. Petkau , T. J. Mitchell , K. Chakraborty , et al., “The Timing of Differentiation and Potency of CD8 Effector Function Is Set by RNA Binding Proteins,” Nature Communications 13 (2022): 2274, 10.1038/s41467-022-29979-x. PMC9046422 35477960

[14] L. S. Matheson , G. Petkau , B. Sáenz‐Narciso , et al., “Multiomics Analysis Couples mRNA Turnover and Translational Control of Glutamine Metabolism to the Differentiation of the Activated CD4+ T Cell,” Scientific Reports 12 (2022): 19657, 10.1038/s41598-022-24132-6. 36385275 PMC9669047

[15] G. Petkau , T. J. Mitchell , M. J. Evans , L. Matheson , F. Salerno , and M. Turner , “Zfp36l1 Establishes the High‐Affinity CD8 T‐Cell Response by Directly Linking TCR Affinity to Cytokine Sensing,” European Journal of Immunology 54 (2024): 2350700, 10.1002/eji.202350700. 38039407 PMC11146077

[16] N. D. Zandhuis , A. Bradarić , Z. C. V. der , A. J. Hoogendijk , B. Popović , and M. C. Wolkers , “Combined Deletion of ZFP36L1 and ZFP36L2 Drives Superior Cytokine Production in T Cells at the Cost of Cell Fitness,” bioRxiv. (2024), 10.1101/2024.12.11.627889. PMC12007392 40249077

[17] M. E. Cook , T. R. Bradstreet , A. M. Webber , et al., “The ZFP36 Family of RNA Binding Proteins Regulates Homeostatic and Autoreactive T Cell Responses,” Science Immunology 7 (2022): eabo0981, 10.1126/sciimmunol.abo0981. 36269839 PMC9832469

[18] M. Turner , B. Sáenz‐Narciso , S. Bell , L. Matheson , and R. Venigalla , “ZFP36‐Family RNA‐Binding Proteins in Regulatory T Cells Reinforce Immune Homeostasis,” Research Square (2024), 10.21203/rs.3.rs-5039504/v1. PMC12056042 40328742

[19] B. Popović , B. P. Nicolet , A. Guislain , et al., “Time‐Dependent Regulation of Cytokine Production by RNA Binding Proteins Defines T Cell Effector Function,” Cell Reports 42 (2023): 112419, 10.1016/j.celrep.2023.112419. 37074914 PMC10242446

[20] N. D. Zandhuis , A. Guislain , A. Popalzij , et al., “Regulation of IFN‐γ Production by ZFP36L2 in T Cells Is Time‐Dependent,” European Journal of Immunology 54 (2024): e2451018, 10.1002/eji.202451018. 38980256

[21] M. A. Coelho , C. de Trécesson S , S. Rana , D. Zecchin , et al., “Oncogenic RAS Signaling Promotes Tumor Immunoresistance by Stabilizing PD‐L1 mRNA,” Immunity 47 (2017): 1083–1099.e6, 10.1016/j.immuni.2017.11.016. PMC5746170 29246442

[22] K. M. Deleault , S. J. Skinner , and S. A. Brooks , “Tristetraprolin Regulates TNF TNF‐α mRNA Stability via a Proteasome Dependent Mechanism Involving the Combined Action of the ERK and p38 Pathways,” Molecular Immunology 45 (2008): 13–24, 10.1016/j.molimm.2007.05.017. 17606294

[23] M. Brook , C. R. Tchen , T. Santalucia , et al., “Posttranslational Regulation of Tristetraprolin Subcellular Localization and Protein Stability by p38 Mitogen‐Activated Protein Kinase and Extracellular Signal‐Regulated Kinase Pathways,” Molecular and Cellular Biology 26 (2006): 2408–2418, 10.1128/mcb.26.6.2408-2418.2006. 16508015 PMC1430283

[24] E. Hitti , T. Iakovleva , M. Brook , et al., “Mitogen‐Activated Protein Kinase‐Activated Protein Kinase 2 Regulates Tumor Necrosis Factor mRNA Stability and Translation Mainly by Altering Tristetraprolin Expression, Stability, and Binding to Adenine/Uridine‐Rich Element,” Molecular and Cellular Biology 26 (2006): 2399–2407, 10.1128/mcb.26.6.2399-2407.2006. 16508014 PMC1430282

[25] C. Tiedje , M. D. Diaz‐Muñoz , P. Trulley , et al., “The RNA‐binding Protein TTP Is a Global Post‐Transcriptional Regulator of Feedback Control in Inflammation,” Nucleic Acids Research 44 (2016): 7418–7440, 10.1093/nar/gkw474. 27220464 PMC5009735

[26] V. Sedlyarov , J. Fallmann , F. Ebner , et al., “Tristetraprolin Binding Site Atlas in the Macrophage Transcriptome Reveals a Switch for Inflammation Resolution,” Molecular Systems Biology 12 (2016): MSB156628, 10.15252/msb.20156628. PMC4988506 27178967

[27] S. Maitra , C.‐F. Chou , C. A. Luber , K.‐Y. Lee , M. Mann , and C.‐Y. Chen , “The AU‐Rich Element mRNA Decay‐Promoting Activity of BRF1 Is Regulated by Mitogen‐Activated Protein Kinase‐Activated Protein Kinase 2,” RNA 14 (2008): 950–959, 10.1261/rna.983708. 18326031 PMC2327367

[28] M. Schmidlin , M. Lu , S. A. Leuenberger , et al., “The ARE‐Dependent mRNA‐Destabilizing Activity of BRF1 Is Regulated by Protein Kinase B,” EMBO Journal 23 (2004): 4760–4769, 10.1038/sj.emboj.7600477. 15538381 PMC535089

[29] S. Adachi , M. Homoto , R. Tanaka , et al., “ZFP36L1 and ZFP36L2 Control LDLR mRNA Stability via the ERK–RSK Pathway,” Nucleic Acids Research 42 (2014): 10037–10049, 10.1093/nar/gku652. 25106868 PMC4150769

[30] D. Benjamin , M. Schmidlin , L. Min , B. Gross , and C. Moroni , “BRF1 Protein Turnover and mRNA Decay Activity Are Regulated by Protein Kinase B at the Same Phosphorylation Sites,” Molecular and Cellular Biology 26 (2006): 9497–9507, 10.1128/mcb.01099-06. 17030608 PMC1698544

[31] F. Salerno , N. A. Paolini , R. Stark , L. M. von , and M. C. Wolkers , “Distinct PKC‐Mediated Posttranscriptional Events Set Cytokine Production Kinetics in CD8+ T Cells,” Proceedings of National Academy of Sciences 2017; 114:9677–9682, 10.1073/pnas.1704227114. PMC5594653 28835535

[32] C. G. King , S. Koehli , B. Hausmann , M. Schmaler , D. Zehn , and E. Palmer , “T Cell Affinity Regulates Asymmetric Division, Effector Cell Differentiation, and Tissue Pathology,” Immunity 37 (2012): 709–720, 10.1016/j.immuni.2012.06.021. 23084359 PMC3622938

[33] S. A. Brooks , J. E. Connolly , and W. F. C. Rigby , “The Role of mRNA Turnover in the Regulation of Tristetraprolin Expression: Evidence for an Extracellular Signal‐Regulated Kinase‐Specific, AU‐Rich Element‐Dependent, Autoregulatory Pathway,” Journal of Immunology 172 (2004): 7263–7271, 10.4049/jimmunol.172.12.7263. 15187101

[34] S. Patial , A. D. Curtis , W. S. Lai , et al., “Enhanced Stability of Tristetraprolin mRNA Protects Mice Against Immune‐Mediated Inflammatory Pathologies,” Proceedings of National Academy of Sciences 113 (2016): 1865–1870, 10.1073/pnas.1519906113. PMC4763790 26831084

[35] W. S. Lai , M. J. Thompson , G. A. Taylor , Y. Liu , and P. J. Blackshear , “Promoter Analysis of Zfp‐36 the Mitogen‐Inducible Gene Encoding the Zinc Finger Protein Tristetraprolin (∗),” Journal of Biological Chemistry 270 (1995): 25266–25272, 10.1074/jbc.270.42.25266. 7559666

[36] O. Bensaude , “Inhibiting Eukaryotic Transcription. Which Compound to Choose?,” Transcription 2 (2011): 103–108, 10.4161/trns.2.3.16172. How to evaluate its activity?. 21922053 PMC3173647

[37] A. R. Clark and J. L. E. Dean , “The Control of Inflammation via the Phosphorylation and Dephosphorylation of Tristetraprolin: A Tale of Two Phosphatases,” Biochemical Society Transactions 44 (2016): 1321–1337, 10.1042/bst20160166. 27911715 PMC5095909

[38] S. Scinicariello , A. Soderholm , M. Schäfer , et al., “HUWE1 controls Tristetraprolin Proteasomal Degradation by Regulating Its Phosphorylation,” Elife 12 (2023): e83159, 10.7554/elife.83159. 36961408 PMC10038661

[39] M. Florkowska , P. Tymoszuk , A. Balwierz , et al., “EGF Activates TTP Expression by Activation of ELK‐1 and EGR‐1 Transcription Factors,” BMC Molecular Biology 13 (2012): 8, 10.1186/1471-2199-13-8. 22433566 PMC3342124

[40] P. Costello , R. Nicolas , J. Willoughby , B. Wasylyk , A. Nordheim , and R. Treisman , “Ternary Complex Factors SAP‐1 and Elk‐1, but Not Net, Are Functionally Equivalent in Thymocyte Development,” Journal of Immunology 185 (2010): 1082–1092, 10.4049/jimmunol.1000472. 20554967

[41] R. Dreos , G. Ambrosini , R. C. Périer , and P. Bucher , “The Eukaryotic Promoter Database: Expansion of EPDnew and New Promoter Analysis Tools,” Nucleic Acids Research 43 (2015): D92–D96, 10.1093/nar/gku1111. 25378343 PMC4383928

[42] T. Sugimoto , S. Stewart , and K.‐L. Guan , “The Calcium/Calmodulin‐Dependent Protein Phosphatase Calcineurin Is the Major Elk‐1 Phosphatase*,” Journal of Biological Chemistry 272 (1997): 29415–29418, 10.1074/jbc.272.47.29415. 9367995

[43] J. Tian and M. Karin , “Stimulation of Elk1 Transcriptional Activity by Mitogen‐Activated Protein Kinases Is Negatively Regulated by Protein Phosphatase 2B (Calcineurin)*,” Journal of Biological Chemistry 274 (1999): 15173–15180, 10.1074/jbc.274.21.15173. 10329725

[44] P. G. Hogan , L. Chen , J. Nardone , and A. Rao , “Transcriptional Regulation by Calcium, Calcineurin, and NFAT,” Genes & Development 17 (2003): 2205–2232, 10.1101/gad.1102703. 12975316

[45] F. Macián , C. García‐Rodríguez , and A. Rao , “Gene Expression Elicited by NFAT in the Presence or Absence of Cooperative Recruitment of Fos and Jun,” Embo Journal 19 (2000): 4783–4795, 10.1093/emboj/19.17.4783. 10970869 PMC302068

[46] G. J. Martinez , R. M. Pereira , T. Äijö , et al., “The Transcription Factor NFAT Promotes Exhaustion of Activated CD8+ T Cells,” Immunity 42 (2015): 265–278, 10.1016/j.immuni.2015.01.006. 25680272 PMC4346317

[47] J. R. Azzi , M. H. Sayegh , and S. G. Mallat , “Calcineurin Inhibitors: 40 Years Later, Can't Live Without,” Journal of Immunology 191 (2013): 5785–5791, 10.4049/jimmunol.1390055. 24319282

[48] J. Aramburu , M. B. Yaffe , C. López‐Rodríguez , L. C. Cantley , P. G. Hogan , and A. Rao , “Affinity‐Driven Peptide Selection of an NFAT Inhibitor More Selective than Cyclosporin A,” Science 285 (1999): 2129–2133, 10.1126/science.285.5436.2129. 10497131

[49] S. Matsuda , T. Moriguchi , S. Koyasu , and E. T. Nishida , “Lymphocyte Activation Signals for Interleukin‐2 Production Involve Activation of MKK6‐p38 and MKK7‐SAPK/JNK Signaling Pathways Sensitive to Cyclosporin A*,” Journal of Biological Chemistry 273 (1998): 12378–12382, 10.1074/jbc.273.20.12378. 9575191

[50] J. Colgan , M. Asmal , B. Yu , and J. Luban , “Cyclophilin A‐Deficient Mice Are Resistant to Immunosuppression by Cyclosporine,” Journal of Immunology 174 (2005): 6030–6038, 10.4049/jimmunol.174.10.6030. 15879096

[51] A. V. Villarino , S. D. Katzman , E. Gallo , et al., “Posttranscriptional Silencing of Effector Cytokine mRNA Underlies the Anergic Phenotype of Self‐Reactive T Cells,” Immunity 34 (2011): 50–60, 10.1016/j.immuni.2010.12.014. 21236706 PMC3955755

[52] M. J. van Stipdonk , G. Hardenberg , M. S. Bijker , E. E. Lemmens , N. M. Droin , D. R. Green , and S. P. Schoenberger , “Dynamic programming of CD8+ T lymphocyte responses,” Nat Immunol 4 (2003): 361, 10.1038/ni912. 12640451

