# Supplementary material for: Differential Regulation of TCR‐Induced ZFP36 and ZFP36L1 Expression by Cyclosporin A in CD8+ T Cells
Source: Eur J Immunol. 2025 Nov 10;55(11):e70085. doi: 10.1002/eji.70085 (PMC12599133; doi:10.1002/eji.70085)
Supplement: Supplementary file 1 — Supporting file 1: eji70085‐sup‐0001‐SuppMat.pdf [file EJI-55-e70085-s001.pdf]

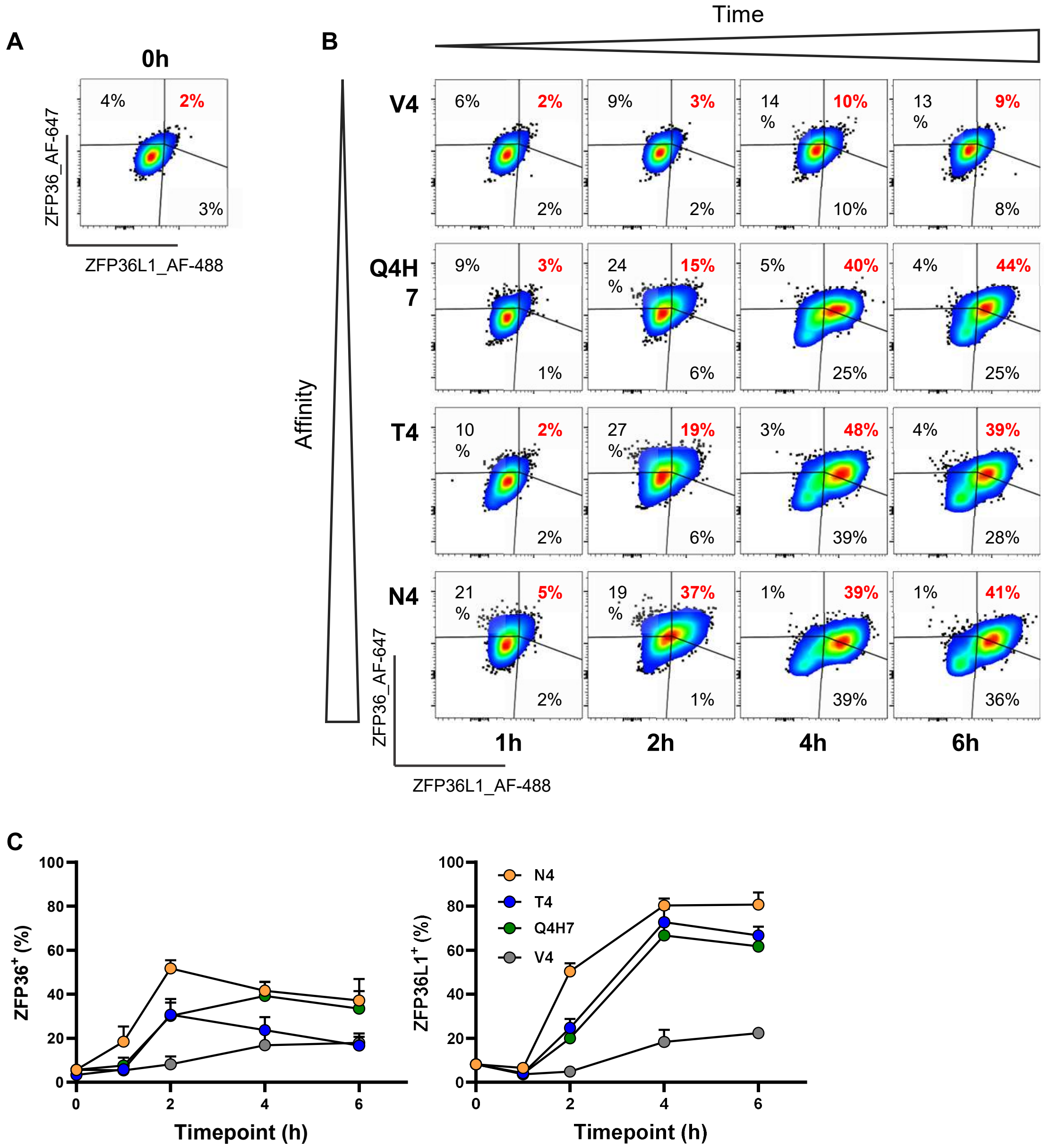
A
0h
4%
2%
3%
ZFP36_AF-647
ZFP36L1_AF-488
B
Time
Affinity
V4
Q4H7
T4
N4
1h
2h
4h
6h
C
ZFP36+ (%)
ZFP36L1+ (%)
Timepoint (h)
N4
T4
Q4H7
V4

Figure S1

Figure S1. ZFP36 and ZFP36L1 expression exhibit distinct kinetics and sensitivity to antigen affinity. Representative flow cytometry plots showing staining with antibodies against ZFP36 and ZFP36L1 in one of three biological replicates of memory-like T cells when (**A**) unstimulated, or (**B**) after stimulation for up to six hours with 0.1 nM N4, T4, Q4H7, or V4. (**C**) The frequencies of ZFP36- and ZFP36L1-expressing cells were plotted; error bars show standard deviation of the mean (SD) of three biological replicates analysed in one experiment.

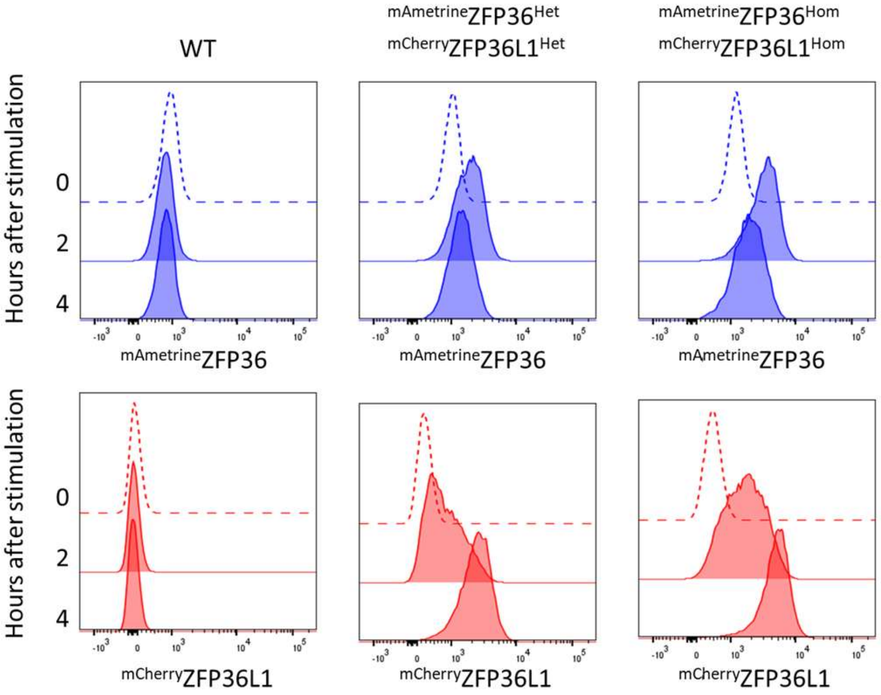
WT
mAmetrineZFP36Het
mCherryZFP36L1Het
mAmetrineZFP36Hom
mCherryZFP36L1Hom
Hours after stimulation
0
2
4
mAmetrineZFP36
mCherryZFP36L1

Figure S2

Figure S2. Homozygous memory T cells are optimal for detection of ^{mAmetrine}ZFP36 and ^{mCherry}ZFP36L1 expression. Wildtype (WT), ^{mAmetrine}ZFP36^{Het} ^{mCherry}ZFP36L1^{Het} or ^{mAmetrine}ZFP36^{Hom} ^{mCherry}ZFP36L1^{Hom} memory-like T cells were stimulated with 0.1 nM N4 for up to four hours for analysis by flow cytometry. Memory-like cells from one biological replicate per genotype was analysed in one experiment.

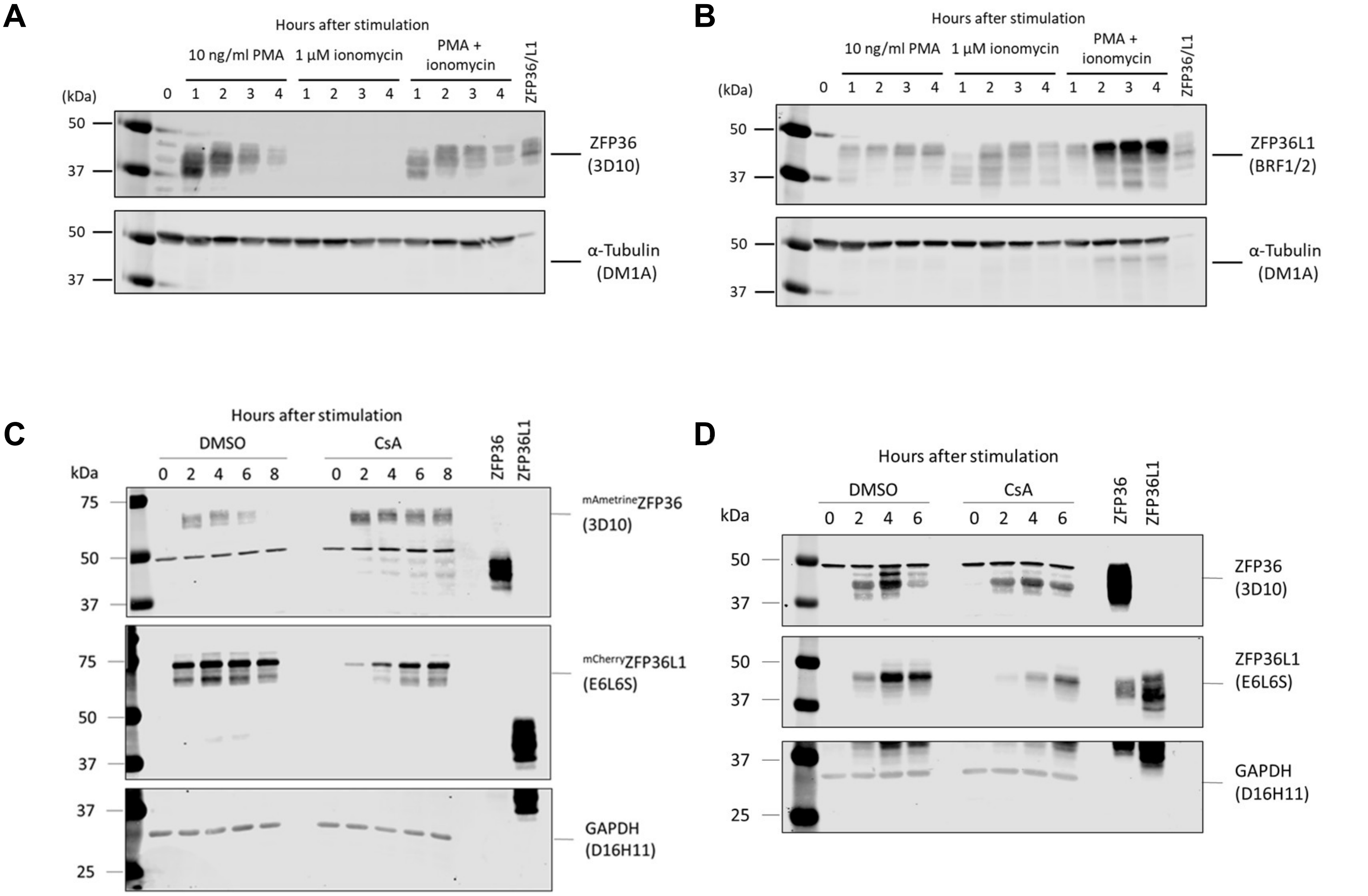
A
Hours after stimulation
10 ng/ml PMA
1 µM ionomycin
PMA + ionomycin
ZFP36/L1
(kDa)
0 1 2 3 4 1 2 3 4 1 2 3 4
50
37
ZFP36 (3D10)
α-Tubulin (DM1A)
B
Hours after stimulation
10 ng/ml PMA
1 µM ionomycin
PMA + ionomycin
ZFP36/L1
(kDa)
0 1 2 3 4 1 2 3 4 1 2 3 4
ZFP36L1 (BRF1/2)
α-Tubulin (DM1A)
C
Hours after stimulation
DMSO
CsA
ZFP36
ZFP36L1
kDa
0 2 4 6 8 0 2 4 6 8
75
50
37
25
mAmetrine ZFP36 (3D10)
mCherry ZFP36L1 (E6L6S)
GAPDH (D16H11)
D
Hours after stimulation
DMSO
CsA
ZFP36
ZFP36L1
kDa
0 2 4 6 0 2 4 6
ZFP36 (3D10)
ZFP36L1 (E6L6S)
GAPDH (D16H11)

Figure S3

Figure S3. Western blot analysis corroborates flow cytometry data that CsA increases ZFP36 and inhibits ZFP36L1 expression. (**A - B**) Wildtype (WT) OT1 memory-like T cells were stimulated for up to four hours with 10 ng/ml PMA and/or 1 µM ionomycin or cultured with DMSO as a control. 30 µg of protein was loaded per lane. (**C**) ^{mAmetrine}ZFP36^{Hom} ^{mCherry}ZFP36L1^{Hom} or (**D**) WT OT1 memory-like T cells were pre-treated with 500 ng/ml CsA or DMSO control before being stimulated with 0.1 nM N4 ± CsA for up to 8 hours. (**C**) 14 µg or (**D**) 20 µg of protein was loaded per lane. (**A - D**) Immunoblots were analysed using anti-ZFP36 (3D10), anti-ZFP36L1 (BRF1/2) or anti-ZFP36L1 (E6L6S), anti-α-tubulin (DM1A) or anti-GAPDH (D16H11) antibodies as indicated. Lysates from HEK 293T expressing FLAG-tagged murine ZFP36 or ZFP36L1 were used as positive controls for blotting where 1 µg of protein was loaded per lane.

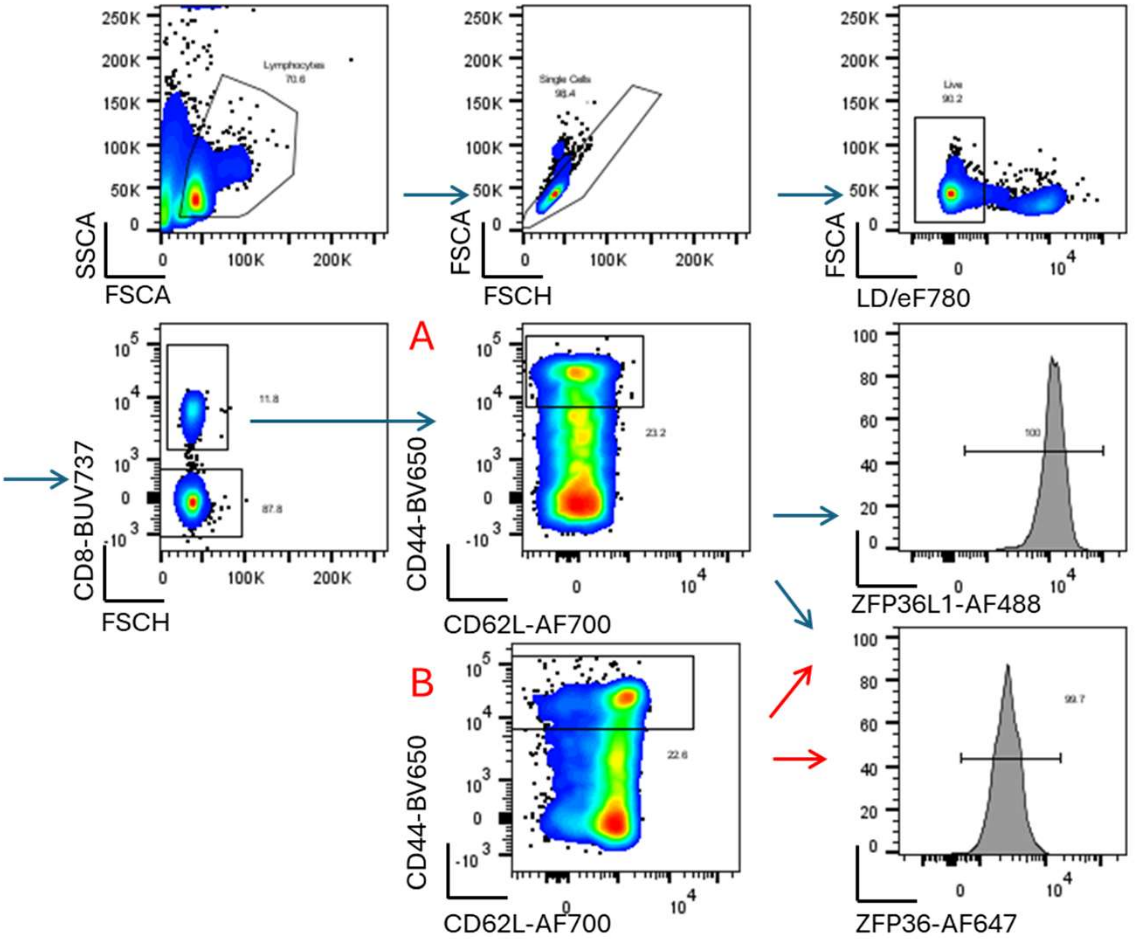

SSCA
FSCA
Lymphocytes
70.6
FSCA
FSCH
Single Cells
98.4
FSCA
LD/eF780
Live
90.2
CD8-BUV737
FSCH
11.8
87.8
A
CD44-BV650
CD62L-AF700
23.2
B
CD44-BV650
CD62L-AF700
22.6
ZFP36L1-AF488
100
ZFP36-AF647
99.7

Figure S4

Figure S4. *Ex vivo* memory T cell flow cytometry gating strategy. Splenocytes from wildtype (WT) mice were gated for analysis of the memory T cell population as follows. The ‘Lymphocytes’ and ‘Single cells’ gate was used to exclude debris and doublets respectively. Live cells were gated according to lack of staining with the viability dye eFluor780. Live CD8^{+} T cells were then gated for CD44^{high} CD62L^{low} expression to identify central memory T cells. ZFP36 and ZFP36L1 expression was analysed in memory T cells (**A**) stimulated for three hours with 10 ng/ml PMA and 1 µM ionomycin and (**B**) unstimulated cells.

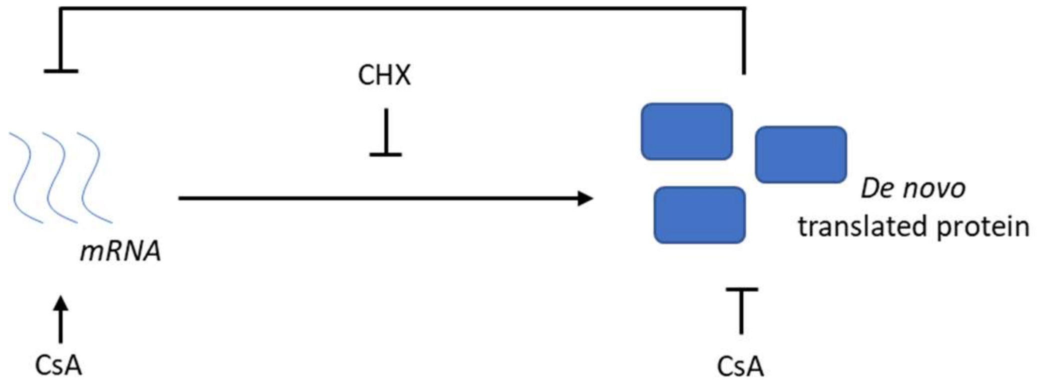

Figure S5

Figure S5. Cycloheximide increases the abundance of mRNA which is negatively regulated by *de novo* translated proteins. For mRNA transcripts that are subject to negative regulation by *de novo* translated proteins, inhibition of translation by cycloheximide results in increased mRNA abundance due to reduced suppression. Regulation of *Zfp36* mRNA by cyclosporin A (CsA) may be the result of increasing the activity of *de novo* translated proteins which suppress *Zfp36* mRNA expression. Alternatively, CsA may act independently of *de novo* protein translation. For example, CsA may promote the transcription of *Zfp36*.

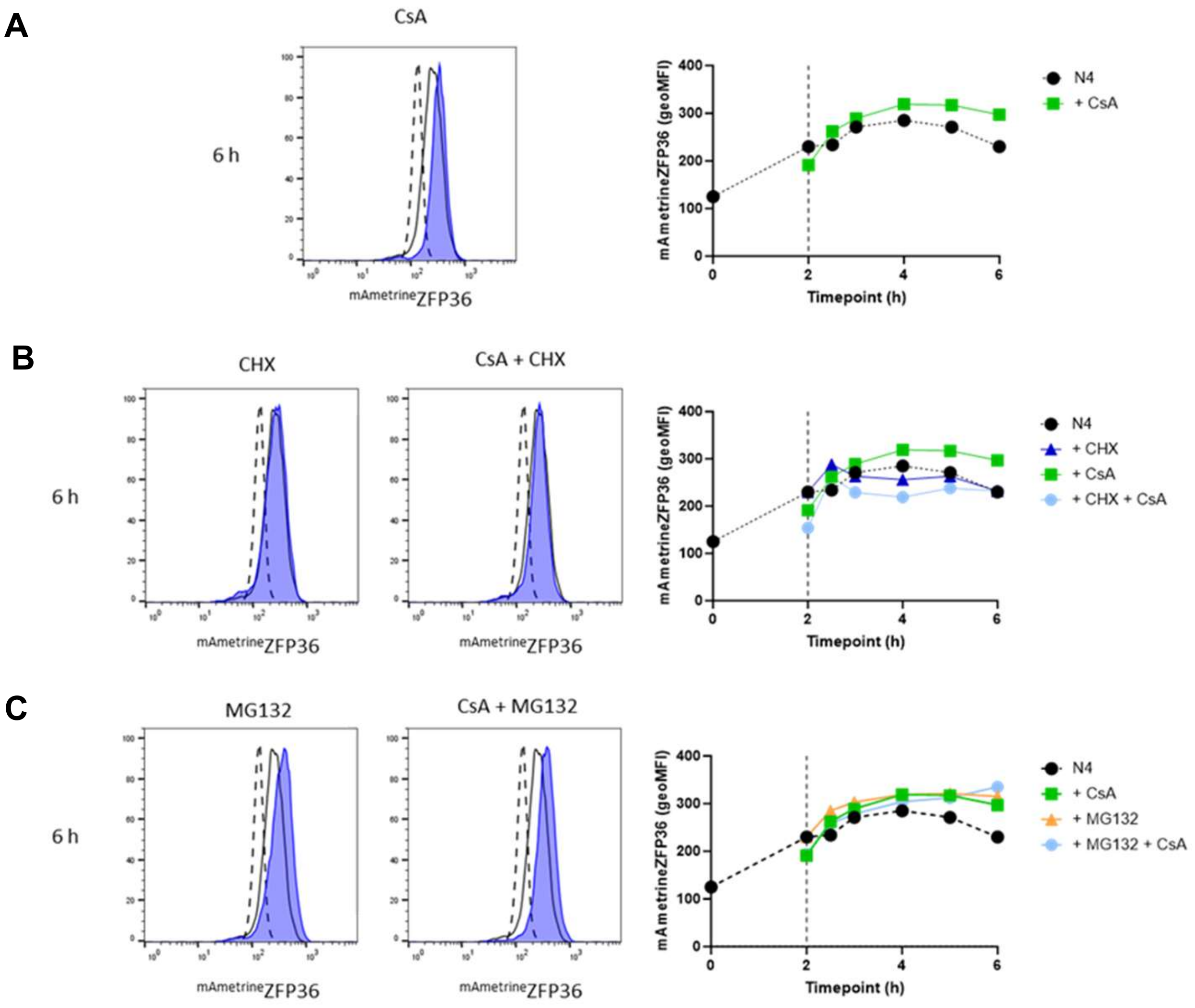
A
CsA
6 h
mAmetrineZFP36
mAmetrineZFP36 (geoMFI)
Timepoint (h)
N4
+ CsA
B
CHX
CsA + CHX
6 h
mAmetrineZFP36
mAmetrineZFP36
mAmetrineZFP36 (geoMFI)
Timepoint (h)
N4
+ CHX
+ CsA
+ CHX + CsA
C
MG132
CsA + MG132
6 h
mAmetrineZFP36
mAmetrineZFP36
mAmetrineZFP36 (geoMFI)
Timepoint (h)
N4
+ CsA
+ MG132
+ MG132 + CsA

Figure S6

Figure S6. Increased ZFP36 protein abundance in the presence of CsA is not due to differences in protein stability or degradation. Memory-like T cells were pre-treated for 30 minutes with 500 ng/ml CsA or DMSO alone before being stimulated with 0.1 nM N4 ± CsA for up to 6 hours. After two hours of stimulation ± CsA, (**B**) 10 µg/ml CHX (translation inhibitor) or (**C**) 10 µM MG-132 (proteasome inhibitor) was added and ^{mAmetrine}ZFP36 expression measured 30, 60, 120, 180 and 240 minutes after the addition of the CHX or MG-132. Graphed data shows ^{mAmetrine}ZFP36 geoMFI of live cells from one homozygous biological replicate analysed in one experiment.

A

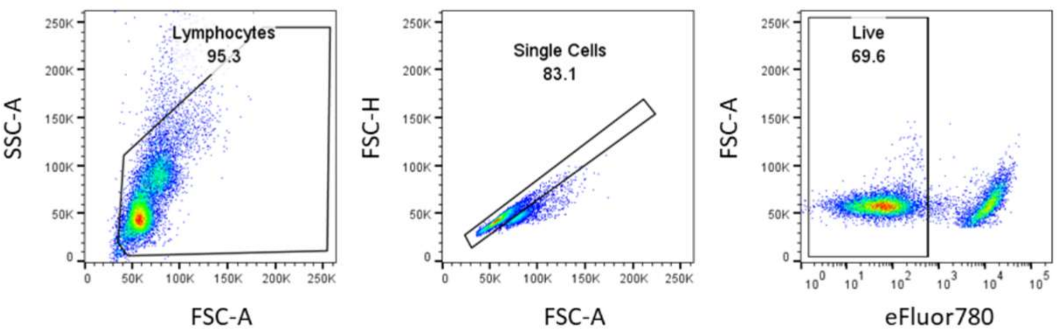
Lymphocytes
95.3
SSC-A
FSC-A
Single Cells
83.1
FSC-H
FSC-A
Live
69.6
FSC-A
eFluor780

B

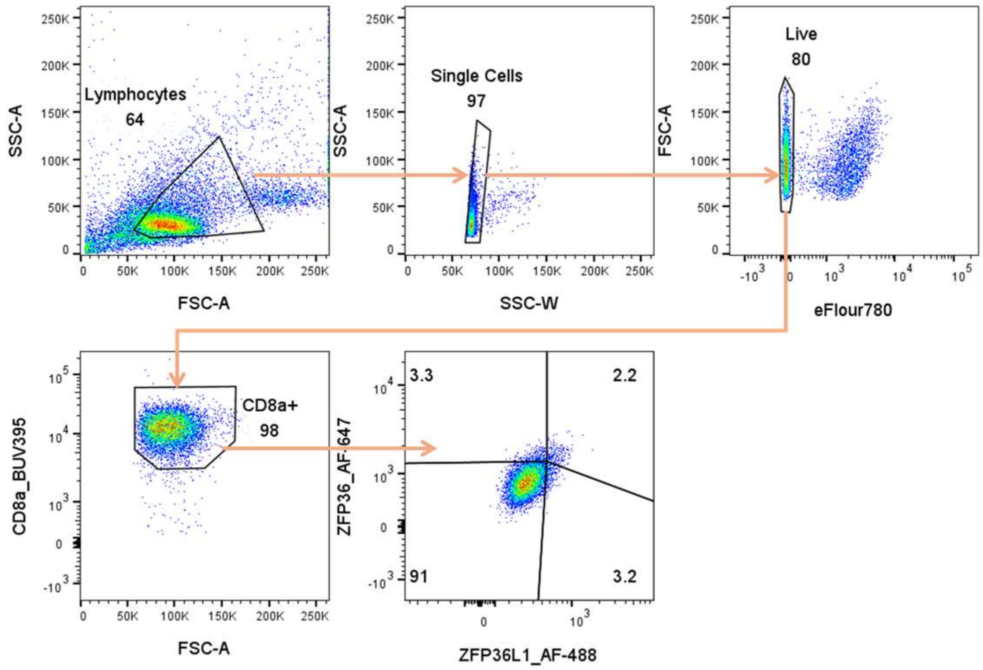
Lymphocytes
64
SSC-A
FSC-A
Single Cells
97
SSC-A
SSC-W
Live
80
FSC-A
eFlour780
CD8a+
98
CD8a_BUV395
FSC-A
3.3
2.2
91
3.2
ZFP36_AF-647
ZFP36L1_AF-488

Figure S7

Figure S7. Representative flow cytometry gating strategy. For analysis of flow cytometry data, the events were first gated to exclude debris and termed ‘lymphocytes’. The events were subsequently gated to exclude doublets, and termed ‘single cells’. Of the single cell events, cells not stained with eFluor780 viability dye were deemed as ‘live’ cells. Where applicable, further subpopulations were gated based on the ‘live’ cell population. (**B**) Single live lymphocytes were gated for CD8 expression for analysis of ZFP36 and ZFP36L1 expression in response to antigen affinity variant OVA peptides. Side scatter area (SSC-A), forward scatter area (FSC-A), forward scatter height (FSC-H).

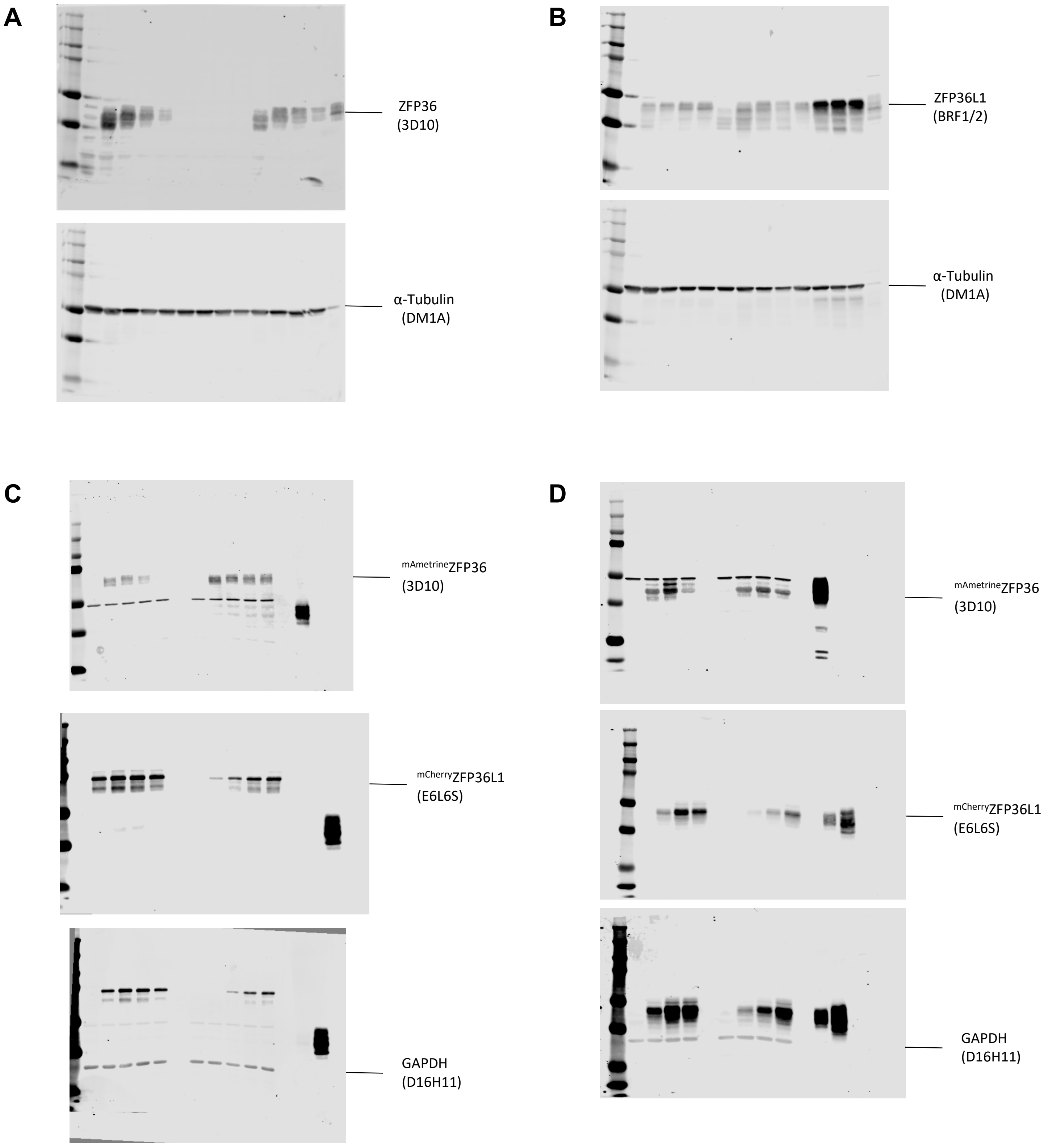

A
ZFP36
(3D10)
α-Tubulin
(DM1A)
B
ZFP36L1
(BRF1/2)
α-Tubulin
(DM1A)
C
mAmetrineZFP36
(3D10)
mCherryZFP36L1
(E6L6S)
GAPDH
(D16H11)
D
mAmetrineZFP36
(3D10)
mCherryZFP36L1
(E6L6S)
GAPDH
(D16H11)

Figure S8

Figure S8. Uncropped Western blot images. Full uncropped images of Western blot membranes presented in **Figure S3** (**A - B**) Wildtype (WT) OT1 memory-like T cells were stimulated for up to four hours with 10 ng/ml PMA and/or 1 µM ionomycin or cultured with DMSO as a control. 30 µg of protein was loaded per lane. (**C**) ^{mAmetrine}ZFP36^{Hom} ^{mCherry}ZFP36L1^{Hom} or (**D**) WT OT1 memory-like T cells were pre-treated with 500 ng/ml CsA or DMSO control before being stimulated with 0.1 nM N4 ± CsA for up to 8 hours. (**C**) 14 µg or (**D**) 20 µg of protein was loaded per lane. (**A - D**) Immunoblots were analysed using anti-ZFP36 (3D10), anti-ZFP36L1 (BRF1/2) or anti-ZFP36L1 (E6L6S), anti-α-tubulin (DM1A) or anti-GAPDH (D16H11) antibodies as indicated. Lysates from HEK 293T expressing FLAG-tagged murine ZFP36 or ZFP36L1 were used as positive controls for blotting where 1 µg of protein was loaded per lane.
